# Med14 phosphorylation shapes genomic response to GLP-1 agonists

**DOI:** 10.1073/pnas.2536772123

**Published:** 2026-03-04

**Authors:** Sam Van de Velde, Jungting Yu, K. Garrett Evensen, Edmund Pakhlevanyan, April E. Williams, Reuben J. Shaw, Marc Montminy

**Affiliations:** ^a^Peptide Biology Laboratories, The Salk Institute for Biological Studies, La Jolla, CA 92037; ^b^Razavi Newman Integrative Genomics and Bioinformatics Core, The Salk Institute for Biological Studies, La Jolla, CA 92037; ^c^Molecular and Cell Biology Laboratory, The Salk Institute for Biological Studies, La Jolla, CA 92037

**Keywords:** GLP-1, PKA, diabetes, transcription, beta cells

## Abstract

Binding of GLP-1 to its receptor in pancreatic beta cells triggers activation of the cAMP pathway and phosphorylation of CREB, leading to induction of cellular target genes containing CREB binding sites. By contrast with their acute effects on beta cell gene expression, chronic exposure of beta cells to stable GLP-1 analogs like Exendin-4 stimulates sustained expression of beta cell-specific genes, leading to increases in beta cell viability and insulin secretion. In a proteomic screen for transcriptional coregulators that contribute to the transcriptional effects of GLP-1, we identified Med14, the scaffolding subunit of the conserved 30 subunit Mediator complex. Exposure to Exendin-4 and other GLP-1 receptor agonists stimulates sustained phosphorylation of Med14 at Ser983, which corresponds to a conserved PKA recognition site. Mutation of Med14 at Ser983 blocked Exendin-4 effects on cellular gene expression by interfering with CREB-mediated activation of beta cell-specific enhancers. Med14 mutation results in higher alpha-to-beta cell ratios and blunted gene regulation in response to Exendin-4 in Ser983-mutant primary mouse islets. Our work reveals how phosphorylation of a general transcription factor in response to GLP-1 analogs triggers a broad genomic response with salutary effects on beta cell function.

## Significance

Stable GLP-1 agonists like Exendin-4 and Ozempic have become a mainstay for treatment of type II diabetes; these peptides enhance insulin secretion and improve beta cell viability in part through sustained activation of the cAMP-CREB pathway. The long-term benefits of GLP-1 receptor agonists appear to reflect the genomic adaptation of beta cells to elevated levels of circulating glucose and lipids in the blood. We found that exposure of pancreatic islet beta cells to GLP-1 receptor agonist promotes phosphorylation of Med14, a subunit of the mediator transcriptional complex. As mutation of Med14 at this phosphorylation site blocks induction of GLP-1-responsive genes, our studies suggest how a general coactivator can promote the selective induction of metabolic programs in response to circulating hormones.

Pancreatic beta cells are equipped to convert metabolic cues of nutrient abundance to insulin secretion. Acute changes in glucose and lipid metabolism during feeding are important in maintaining energy balance; chronic increases in these circulating metabolites disrupt beta cell function and lead to type 2 diabetes. Hyperglycemia stimulates glycolytic and lipogenic gene programs in beta cells through induction of Hypoxia Induced Factor (HIF) and sterol response element binding protein (SREBP) (1–6). These responses promote adaptation to acute increases in circulating glucose concentrations, while also contributing to gluco- and lipotoxicity in the setting of chronic hyperglycemia.

Feeding stimulates release of GLP-1 from enteroendocrine cells of the gut to maintain blood glucose homeostasis through induction of insulin release and suppression of glucagon secretion (7, 8). Triggering of the GLP-1 receptor in beta cells by the stable GLP-1 analog Exendin-4 (Ex-4) increases intracellular cAMP production. Ex-4 cooperates with circulating glucose levels to stimulate insulin release acutely and to increase insulin gene expression and beta cell survival (9). The long-term metabolic benefits of Ex-4 suggest that it promotes a beta cell specific genomic adaptation, which mitigates the response to increased metabolic demands in the setting of insulin resistance (10, 11).

Indeed, Ex-4 appears to act cooperatively with glucose to promote expression of beta cell-specific genes via the induction of CREB and its coactivators. Depletion of CREB family member activity in beta cells through expression of dominant-negative ACREB causes beta cell failure and diabetes (12), while deletion of CREB in adult beta cells blunts Exendin-4 induced insulin secretion and glucose tolerance in high fat diet fed animals (13). However, the mechanisms linking gene regulation by cAMP with the salutary effects of GLP-1 analogs remain poorly understood. In a proteomic screen for transcriptional regulators that are phosphorylated by PKA in response to cAMP signaling in INS-1 beta cells, we identified MED14, a subunit of the Mediator complex.

Mediator is a multisubunit nuclear complex that governs transcription at the level of preinitiation complex (PIC) organization, RNA polymerase II (Pol II) C-terminal domain phosphorylation, Pol II pause release, and enhancer activation (14). Structurally, mediator consists of 30 subunits that are connected by the scaffolding subunit Med14. Mediator also contains a kinase module that controls activity of Pol II and transcriptional elongation factors (15–18). Mediator is thought to trigger the expression of metabolic programs by associating with DNA-specific transcription factors (TFs). TFs that directly bind mediator subunits include a number of metabolic regulators, such as peroxisome proliferator-activated receptor alpha and gamma (PPARα, γ) and SREBP (19–24).

Here, we identify a conserved PKA phosphorylation site in Med14 that promotes sustained beta cell target gene expression, in part by activation of CREB bound enhancers. We find that broad transcriptional regulation of metabolism coincides with cellular lipid imbalance in Med14 S983A mutant INS-1 cells. Med14 S983A mutation also blocks gene regulation by sustained Ex-4 exposure in primary beta cells. Together, our results reveal how GLP-1 analogs reprogram the beta cell genome in part through the phosphorylation of a mediator subunit.

## Results

### Ex-4 Triggers a Robust Transcriptional Response in Beta Cells.

**Fig. 1.**
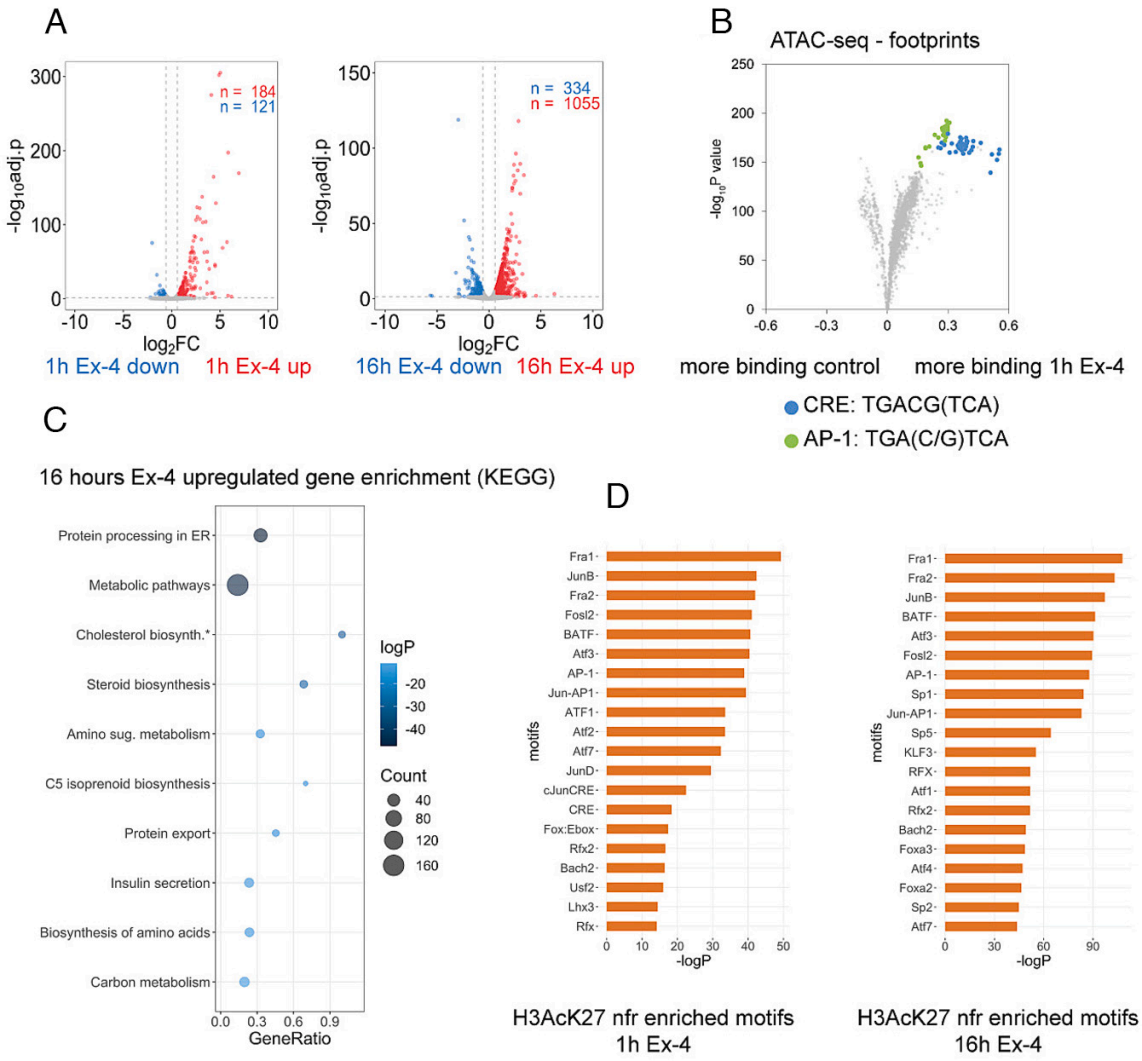
Ex-4 triggers a robust gene response. (*A*) Differential gene expression after acute (1 h, *Left*) or sustained (16 h, *Right*) Ex-4 (10 nM) exposure in INS-1 cells. RNA-seq was performed in duplicate. (*B*) Volcano plot depicting change in footprint score over transcription factor binding motifs in accessible chromatin regions (ATAC) after 1 h Ex-4 (10 nM) treatment. Motifs corresponding to CREB response binding (CRE) and activator protein-1 (AP-1) are highlighted. (*C*) KEGG pathway enrichment analysis of genes induced after 16 h Ex-4 (10 nM) exposure. (*D*) Motifs enriched in nucleosome-free regions (nfr) of active enhancers (H3AcK27 ChIPseq) annotated to genes induced after 1 h (*Left*) and 16 h (*Right*). See also *SI Appendix*, Fig. S1 and Datasets S1–S6.

To investigate the mechanism by which Ex-4 promotes insulin secretion and beta cell viability, we performed Spike-in normalized RNA sequencing (RNA-seq) on INS-1 insulinoma cells upon acute (1 h) or prolonged (16 h) Ex-4 exposure. Ex-4 treatment for one hour induced a limited set of genes (n = 184; FC > 1.5, padj. < 0.05): many of these correspond to core CREB target genes that enriched in feedback inhibition of upstream signaling (e.g., *Pde10a*, *Rgs2*, *Dusp1*, *Sik1*) and activator protein (AP-1) transcription factors (e.g., *Fos*, *JunB*, *Fosl1*, *Fosl2*) (Fig. 1*A* and Dataset S1) (25).

By contrast with these short-term effects, prolonged exposure (16 h) of INS-1 cells to Ex-4 generated a far more robust transcriptional response (n = 1,065; FC > 1.5, padj. < 0.05) (Fig. 1*A* and Datasets S2 and S3). Gene responses to Ex-4 overlapped almost entirely with the adenylate cyclase agonist forskolin (FSK), confirming that Ex-4 promotes gene expression primarily through activation of the cAMP pathway (*SI Appendix*, Fig. S1 *A*–*C* and Datasets S4–S6) (26).

To determine the mechanism by which Ex-4 modulates gene expression we performed ChIP-seq studies of cAMP-responsive factors (CREB, CRTC2), activated histone marker H3AcK27, and elongating phosphorylated RNA polymerase (Pol IIpS2). Inspection of the genomic region surrounding the CREB-target gene *Irs2* revealed comparable CREB/CRTC2 recruitment, enhancer activation, and Pol IIpS2 elongation by Ex-4 and FSK (*SI Appendix*, Fig. S1*D*). To further validate the role of CREB in the genomic response to Ex-4, we conducted in vivo footprinting analysis with high depth (~250x10^6^ reads per sample) assay for transposase-accessible chromatin with sequencing (ATAC-seq) datasets. Footprint scoring in accessible chromatin revealed significant enrichment of CREB and CREB target AP-1 binding motifs in INS-1 cells following short-term treatment with Ex-4 and FSK (Fig. 1*B* and *SI Appendix*, Fig. S1 *E* and *F*). Taken together, these findings confirm the importance of CREB/CRTC2 and AP-1 as drivers of gene expression by Ex-4.

The induction of core CREB target genes in response to Ex-4 is immediate and largely extinguished after 4 to 6 h (27). To investigate the role of sustained gene induction, we performed pathway analysis of transcripts induced after sustained Ex-4 and FSK exposure. Genes involved in protein processing in the Endoplasmic Reticulum (ER), metabolism, protein secretion, and cholesterol biosynthesis were up-regulated following 16 h Ex-4 treatment by KEGG pathway and overrepresentation analysis (ORA) (Fig. 1*C* and *SI Appendix*, Fig. S1*G*). These results are consistent with a potential role for CREB in beta cell adaptation to nutrient imbalance. Motif analysis of nucleosome-free regions (NFRs) in H3AcK27 decorated active enhancers annotated to Ex-4 induced genes revealed enrichment of cAMP response elements (CREs) and AP-1 motifs after acute and sustained Ex-4 treatment (Fig. 1*D*), in agreement with earlier work (28). These findings suggest that CREB and AP-1 transcription factors play a role in both immediate and delayed gene responses to Ex-4.

### Med14 Is a PKA Target.

**Fig. 2.**
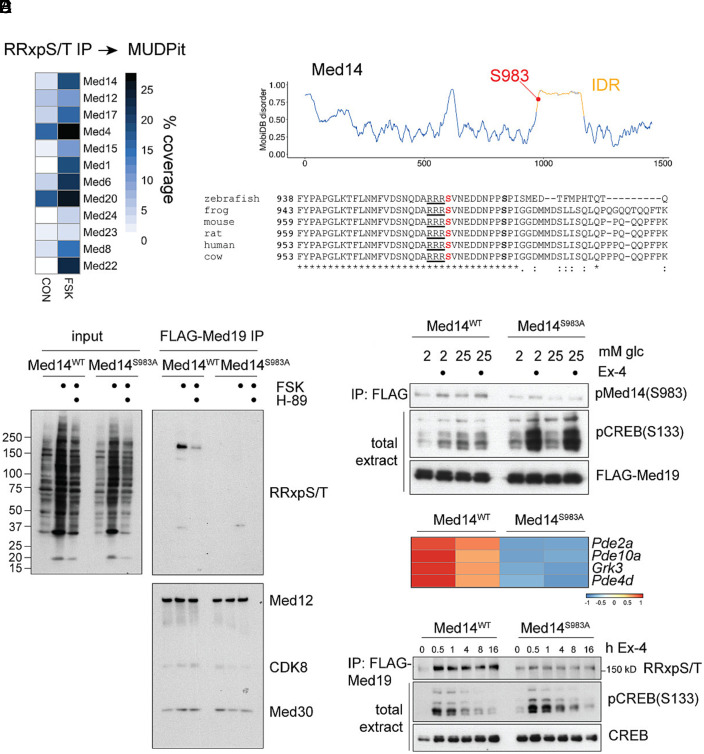
Med14 is a PKA target. (*A*) MUDPit coverage of mediator complex subunits recovered in PKA substrate (RRxpS/T) immunoprecipitates from control and FSK (10 μM) treated cell lysates. (*B*) MobiDB disorder score over Med14. S983 is indicated inside the IDR (*Top*). Alignment of Med14 orthologs highlighting conservation of the PKA substrate motif and the Erk target site Ser992 (*Bottom*). (*C*) Western blot of PKA substrate antibody on input (*Left*) and endogenous FLAG-Med19 immunoprecipitates (IP) (*Right*) in Med14WT and Med14 S983A Crispr knock-in lines. Integrity of mediator complex in IP is probed by Med12, Cdk8, and Med30 blots. PKA activation by FSK and inhibition by H-89 is shown in total extract. (*D*) Med14 S983 and CREBS133 phosphorylation by Ex-4 (10 nM) in WT and Med14 S983A mutant lines. FLAG-Med19 is probed as loading control in total extract. (*E*) Expression of cAMP signaling pathway component genes is repressed in Med14 S983A mutant. (*F*) Time-course western blot after treatment of WT and Med14 S983A cells with Ex-4 (10 nM). FLAG-Med19 immunoprecipitates are probed with PKA substrate (RRxpS/T) antibody. Phosphorylated (pS133) and total CREB are probed in total extract. See also *SI Appendix*, Fig. S2.

We performed a proteomic screen of INS-1 cell extracts to identify regulatory proteins that are phosphorylated by PKA and that may function in modulating the transcriptional response to Ex-4. We recovered multiple Mediator subunits in Phospho-PKA substrate (RRxpS/T) antibody immunoprecipitates of INS-1 cells exposed to FSK by mass spectrometry (Fig. 2*A*). Of the 10 identified Mediator components, only the scaffolding subunit Med14 was found to contain a conserved PKA substrate motif at Ser983 (in the mouse) (Fig. 2*B*). Ser983 is located within a 200 amino acid intrinsically disordered region (IDR) near the C-terminal tail of Med14, a region that connects the core and tail sections of mammalian Mediator (*SI Appendix*, Fig. S2*A*) (15, 16).

To address whether Med14 S983 is phosphorylated by PKA, we generated independent Med14 Ser983Ala mutant INS-1 lines (see methods). We also generated endogenous FLAG-Strep-Med19 fusions in WT and Med14 S983A INS-1 backgrounds to isolate WT and mutant Mediator complexes. FLAG-Med19 immunoprecipitates from WT and Med14S983A extracts followed by immunoblot with phospho-PKA substrate antibody confirmed phosphorylation around the predicted molecular weight of Med14 (~160 kD) in WT, but not mutant Med14 cells. Moreover, Med14 phosphorylation was blocked by the PKA inhibitor H-89 (Fig. 2*C*).

In principle, Med14 S983 mutation could destabilize Mediator and disrupt interaction of Med19 with other Mediator subunits, thereby impeding detection of phospho-PKA substrate immunoreactivity in other Mediator constituents or interacting TFs. Arguing against this notion, immunoblot for additional Mediator subunits (MED12, CDK8, and MED30) in FLAG-Med19 immunoprecipitates revealed comparable amounts of these proteins in WT and Med14 S983A mutant complexes (Fig. 2*C*). We further confirmed the integrity of Med14 S983A mutant Mediator by glycerol gradient sedimentation of FLAG-Med19 immunoprecipitates (*SI Appendix*, Fig. S2*B*). In addition to its effects on PKA activity, cAMP is also known to activate the growth factor–dependent kinase Erk in beta cells, which could lead to phosphorylation of known Erk target sites adjacent to Ser983 in Med14 (29). Phosphorylation of these sites, most notably Ser992, could influence Ser983 phosphorylation in a PKA-independent manner. We tested this possibility by activating Erk signaling through growth factor stimulation of serum starved HEK293T cells, and by overexpression of the active kRas(G12V) allele in cells expressing HA-tagged Med14 S983A and S992A mutants. These experiments showed that, while growth factor signaling appeared to alter Med14 protein stability, Ser983 phosphorylation was independent of Ser992 arguing against a role for Erk stimulation in this context (*SI Appendix*, Fig. S2 *C* and *D*). Taken together, these findings show that Med14 is the predominant PKA substrate in the Mediator complex.

To determine whether Ex-4 modulates Med14 phosphorylation, we generated a phospho-specific antibody targeting the Med14pS983 site. In the basal state, WT and S983A mutant Med14 were undetectable by immunoblot using the phospho-specific antiserum. Ex-4 treatment increased amounts of phospho-Med14 in WT but not mutant Med14 in both low (2 mM) and high (25 mM) glucose media. By contrast, exposure of Med14 S983A cells to Ex-4 strongly induced CREB hyperphosphorylation in response to Ex-4, suggesting that Med14, like CREB, plays a critical role in regulation of upstream cAMP signaling (Fig. 2*D*). Among the genes repressed in Med14S 983A lines, we identified phosphodiesterases (*Pde2a*, *Pde10a*, *Pde4d*), as well as the G-protein-coupled receptor kinase *Grk3*, which controls GLP-1 receptor activity and ligand-induced receptor internalization (Fig. 2*E*) (30, 31). Consistent with the notion that CREB activates immediate-early gene expression, stimulus-induced CREB phosphorylation decreases upon prolonged stimulation through dephosphorylation by PP1 and PP2A (32, 33). By contrast, phosphorylation of Med14 by Ex-4 was sustained, even after 16 h stimulation (Fig. 2*F*). Together, these results suggest that phosphorylation of CREB and Med14 cooperatively promote gene induction by Ex-4 on different timescales.

### Med14 S983 Phosphorylation Promotes Target Gene Induction Through Activation of Relevant Enhancers.

**Fig. 3.**
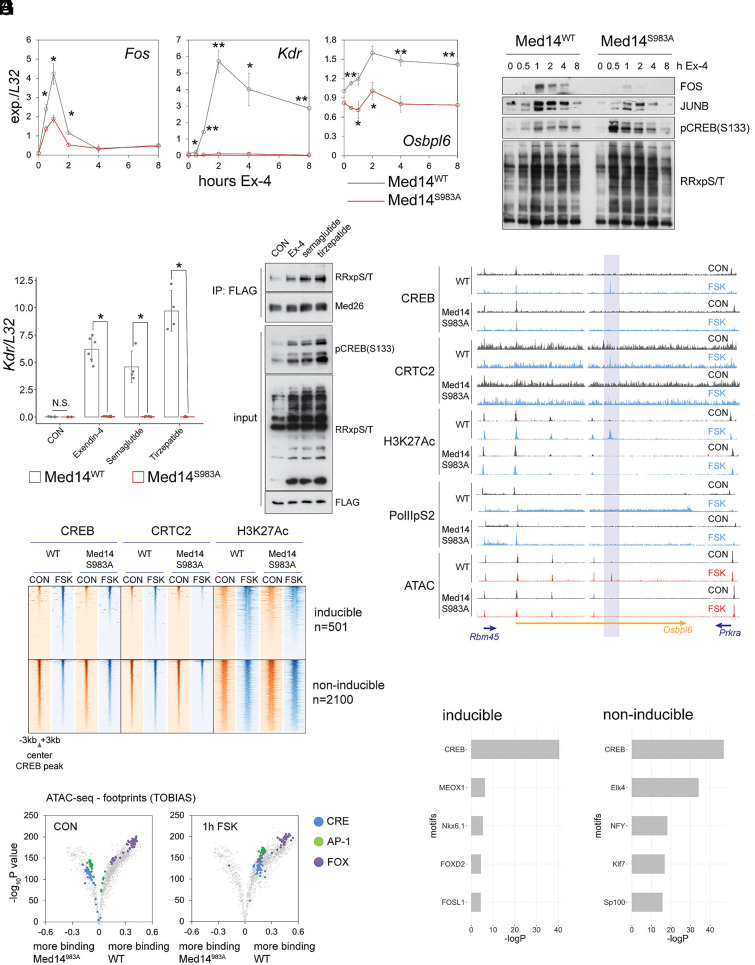
Med14 S983 phosphorylation promotes gene induction by activating enhancers. (*A*) Time course Q-PCR for immediate-early (*Fos*, *P < 0.02) and delayed-early (*Kdr*, *P < 0.02, **P < 8E-4), (*Osbpl6*, *P < 0.05, **P < 0.007) genes over 8 h Ex-4 (10 nM) treatment in WT and Med14 S983A mutant cells. Error bars show SD. Two-way ANOVA with Bonferroni post hoc analysis. (*B*) Time course western blot for core CREB target gene products Fos and JunB, phospho-CREB(S133), and phospho-PKA target over 8 h Ex-4 (10 nM) treatment. (*C*) Q-PCR for induction of *Kdr* by three GLP-1 agonists: Exendin-4, Semaglutide, and Tirzepatide (10 nM) in Med14 WT and Med14 S983A mutant INS-1 cells. Cells were treated for 2 h. Error bars show SD. *P < 5E−5 by the unpaired *t* test. (*D*) Western blot showing Med14 phosphorylation by Exendin-4, Semaglutide, and Tirzepatide (10 nM) in FLAG-Med19 immunoprecipitates. Med26 is shown to confirm equal mediator quantity in IPs. CREB phosphorylation, PKA activation, and FLAG-Med19 levels are shown in total extract. (*E*) Chromatin occupancy (ChIP-seq) of CREB, CRTC2 and H3AcK27 in WT and Med14 S983A mutants in basal and FSK-stimulated conditions over cAMP-inducible (*Top*) and non-inducible (*Bottom*) enhancers. See also *SI Appendix*, Fig. S3 and Datasets S7 and S8. (*F*) Example of an inducible enhancer in an *Osbpl6* intron. Tracks are scaled across conditions for each ChIP target and for ATAC. Intronic cAMP responsive enhancer is highlighted. (*G*) Volcano plot comparing change in binding over known transcription factor binding motifs between WT and Med14 S983A mutants in control (left) and FSK (10 μM) treated (right) cells. Footprinting analysis was performed on high-depth (~250x106 reads per sample) ATAC-seq data. (*H*) Enrichment of transcription factor binding motifs over cAMP-inducible (*Left*) and non-inducible (*Right*) enhancers. See also *SI Appendix*, Fig. S3.

We next explored the role of Med14 phosphorylation in promoting Ex-4-induced gene expression. RT-QPCR analysis of core CREB target genes in WT and Med14 S983A mutant INS-1 lines revealed that *Fos* induction peaked at both protein and mRNA levels after 1 h and returned to baseline within 4 to 8 h at both mRNA and protein levels (Fig. 3 *A* and *B*). Further, Ex-4- and FSK-induced activity of a CRE-luciferase reporter was blunted when expressed in Med14 S983A mutant cells (*SI Appendix*, Fig. S3*A*). Notably, delayed-early gene (DEG) induction (*Kdr*, *Osbpl6*) was quantitatively blocked in Med14 S983A mutant lines exposed to Ex-4 (Fig. 3*A*). To further confirm the role of GLP-1 agonists and cAMP in mediating these effects, we repeated gene induction of DEGs using the clinically relevant peptides Semaglutide and Tirzepatide as well as FSK. *Kdr* gene induction, PKA activity, and Med14 phosphorylation were similarly induced by Ex-4, Semaglutide, and Tirzepatide (Fig. 3 *C* and *D*). Moreover, *Kdr*, *Osbpl6,* and tissue-type plasminogen activator (*Plat*) genes were substantially up-regulated following activation of the cAMP-PKA pathway in INS-1 cells by FSK (*SI Appendix*, Fig. S3*B*). In contrast, triggering of the NFκB pathway in response to IL-1β actually increased in Med14 S983A mutant cells relative to WT, supporting the notion that Med14 phosphorylation has distinct effects on cAMP inducible and proinflammatory genes (*SI Appendix*, Fig. S3*C*).

cAMP-inducible gene expression is dependent on CREB occupancy over stimulus-responsive promoter-distal enhancers, which are defined by corecruitment of CBP/p300 and subsequent activating acetylation of histones H3 and H2A.Z (34, 35). Of the 4200 CREB bound enhancer regions in INS-1 cells, only ~12% (501) exhibited increases in H3AcK27 following exposure to FSK, suggesting that additional nuclear factors are required for stimulus-induced transcription (Datasets S7 and S8). Mediator has been found to promote transcription from tissue-specific rather than global enhancers, prompting us to consider that CREB and Med14 act cooperatively on Mediator sensitive enhancers following their phosphorylation by PKA. Supporting this idea, inducible enhancer activity at CREB bound genomic loci was significantly reduced in Med14 S983A mutant cells relative to WT. ChIPseq data for H3AcK27 revealed a reduction in inducible enhancer activity in MED14 S983A mutant INS-1 cells, while enhancer activity was unchanged either globally or across noninducible CREB bound enhancers. In line with this result, CREB and CRTC2 occupancy in Med14 S983A mutant cells are decreased over inducible enhancers in response to FSK (Fig. 3 *E* and *F* and *SI Appendix*, Fig. S3*D*).

Tissue-specific enhancers exhibit enhanced nucleosome clearance compared to ubiquitous regulatory regions in the genome (36). We examined the contribution of Med14 phosphorylation on DNA accessibility in enhancers by ATAC-seq. As expected, 1 h Ex-4 or FSK treatment increased chromatin accessibility over cAMP responsive, but not noninducible enhancers (Fig. 3*D* and *SI Appendix*, Fig. S3 *E* and *F*). Stimulus-induced accessibility was blunted in Med14 S983A mutant cells. Genome-wide footprinting analysis on ATAC-seq data revealed significant enrichment of CREB binding motifs after acute Ex-4 or FSK stimulation, consistent with a role for CREB in mediating the immediate early gene response to cAMP (Fig. 3*F* and *SI Appendix*, Fig. S3*F*). Surprisingly, binding over CRE and AP-1 motifs was enriched in Med14 S983A relative to WT cells under basal conditions by footprinting analysis. In line with the role of forkhead pioneering factors and mediator in activating tissue-specific enhancer regions, FOXO motifs were enriched in open chromatin of WT cells and over cAMP inducible enhancer regions (Fig. 3*G*).

### Med14 S983 Is a Critical Mediator of Insulin Gene Expression.

**Fig. 4.**
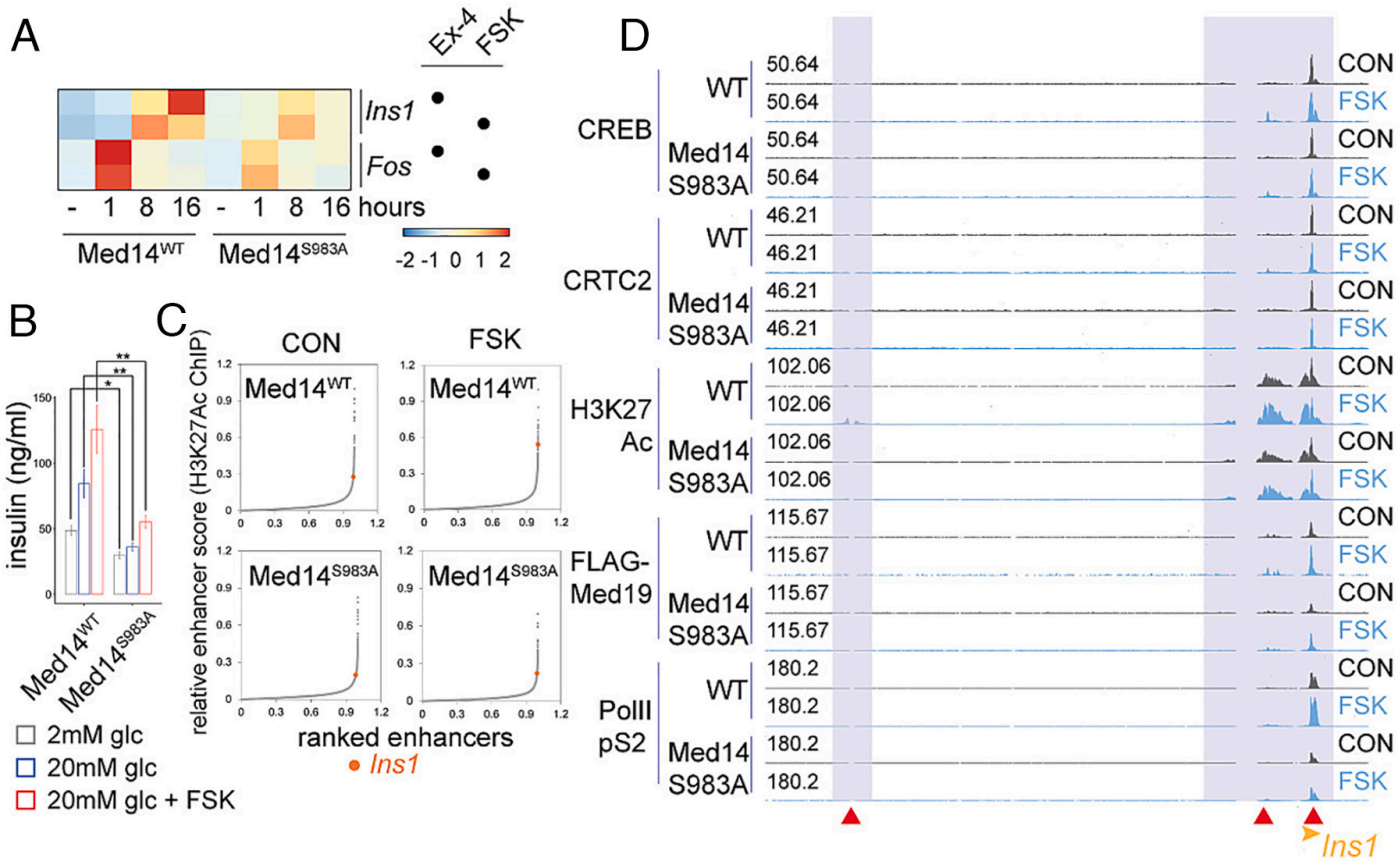
Med14S983 is a critical mediator of insulin gene induction. (*A*) Induction profile of *Ins1* and *Fos* by Ex-4 (10 nM) or FSK (10 μM) in WT and Med14 S983A mutants. (*B*) Insulin secretion in WT and Med14 S983A mutant cells. Cells were stimulated for 1 h with glucose in the indicated concentrations and FSK (10 μM). *P < 0.02, **P < 0.0001. One-way ANOVA with Holm-Sidak post hoc test. This test was performed on triplicates for each sample and was repeated with an independently generated Med14 S983A line. (*C*) Ranked enhancer score (H3AcK27 ChIPseq) plots of WT (*Top*) and Med14 S983A mutant cells (*Bottom*) in basal or FSK stimulated state. Insulin (*Ins1*) is highlighted. (*D*) ChIPseq tracks at the *Ins1* locus in basal and FSK stimulated states comparing ChIP occupancy between WT and Med14 S983A mutant cells.

Insulin is the most highly expressed cell-specific gene in beta cells and its induction by cAMP is thought to promote beta cell adaptation to increases in circulating glucose and fatty acids. By contrast with the rapid induction of the *Fos* gene (1 h) by Ex-4 and FSK, *Ins1* gene expression peaked after 16 h in response to both stimuli; these effects were blunted in Med14 S983A mutant cells (Fig. 4*A*). In line with this observation, basal and stimulus-induced insulin secretion was blunted in the Med14 S983A mutant (Fig. 4*B*). Indeed, ranked enhancer strength plots (see methods) revealed promotion of relative *Ins1* enhancer activity upon FSK exposure in WT but not Med14 S983A mutant cells, indicating that Med14 S983 phosphorylation may be required for stimulus-induced *Ins1* expression through enhancer activation (Fig. 4*C*). Further inspection of the *Ins1* locus showed reduced activation of two proximal enhancers, as well as decreased recruitment of CREB, CRTC2, and Mediator over proximal genomic regions (Fig. 4*D*).

### Ex-4 Tunes Metabolism Through Med14.

**Fig. 5.**
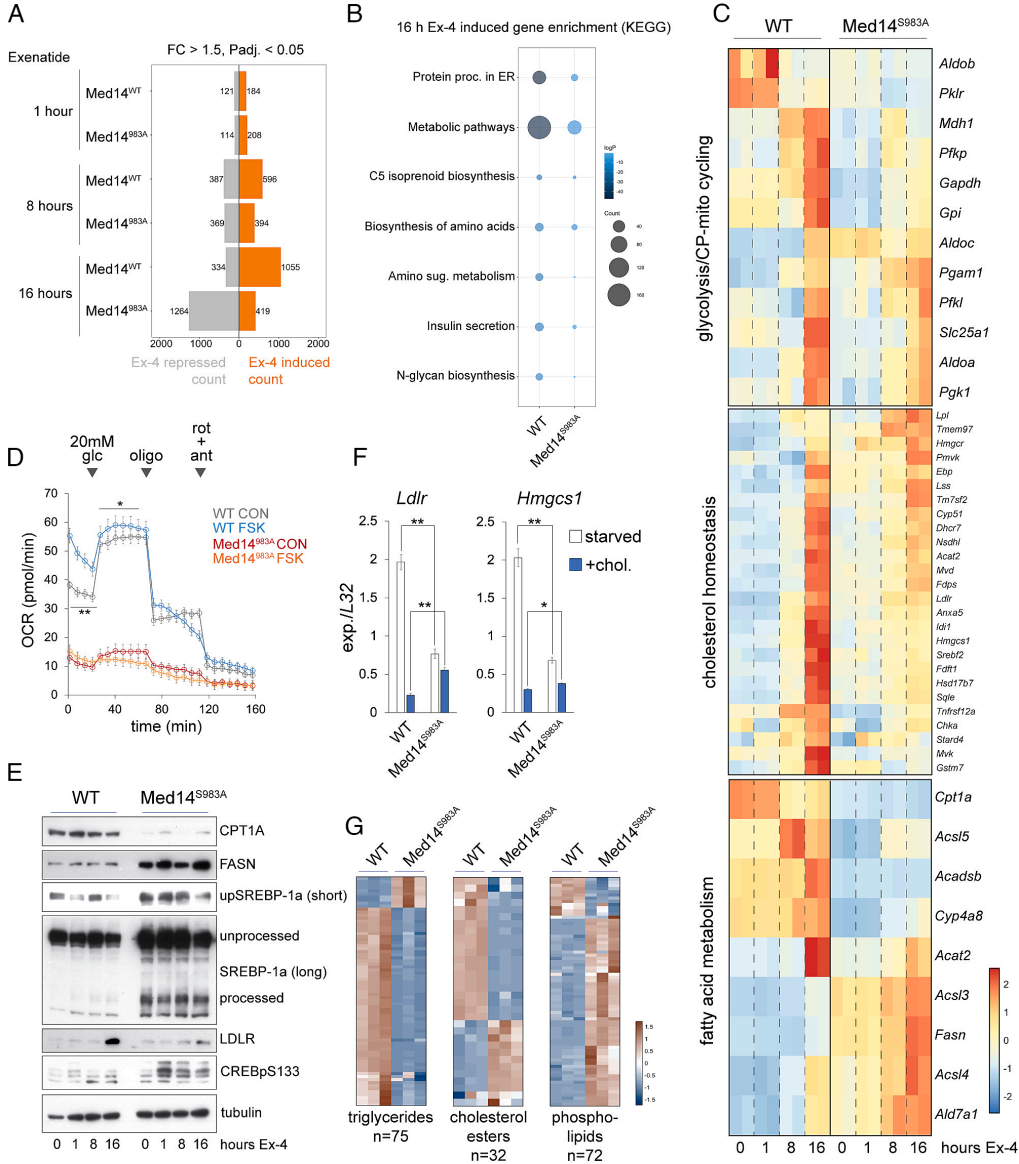
Ex-4 tunes metabolism through Med14. (*A*). Ex-4 regulated gene counts from spike-in corrected RNA-seq after 1, 8, and 16 h exposure in WT and Med14 S983A mutant cells. (*B*). KEGG pathway enrichment of Ex-4-induced genes (16 h) in WT and Med14 S983A mutant cells. (*C*). Heatmaps of metabolic gene regulation by Ex-4. Glycolytic genes with *Mdh1* and *Slc25a1* (*Top*), cholesterol homeostasis genes (*Middle*), and fatty acid metabolism genes (*Bottom*). (*D*). Oxygen consumption rate (OCR) of WT and Med14 S983A mutants with or without 16 h pre-exposure to FSK (10 uM). Basal glucose concentration is 2 mM. Cells were treated with 20 mM glucose, 5 uM oligomycin and 5 uM rotenone + 5 uM antimycin A at the indicated timepoints. This test was performed in 10-12 wells of a 96-well Seahorse assay plate per sample. Each test was repeated with an independently generated Med14 S983A line. Statistical significance was calculated between control and FSK treated WT cells by Two-way ANOVA with Bonferroni post hoc analysis. *P < 7E−7. (*E*). Western blots probing fat metabolism and CREB activation in WT and Med14 S983A mutant cells. Cells were exposed to Ex-4 as indicated. Unprocessed and cleaved SREBP-1a bands are marked. (*F*). RT-qPCR of *Ldlr* and *Hmgcs1* comparing WT and Med14 S983A mutant cells. Cells were cultured for 4 d in lipoprotein deficient serum and treated with 10 μg/mL cholesterol plus 1 μg/mL 25-hydroxycholesterol for 8 h as indicated. *P < 0.0003 by one-way ANOVA with Holm-Sidak post hoc test. (*G*). Heatmap comparing levels of triglycerides (*Left*), cholesterol esters (*Middle*) and phospholipids (*Right*) in WT and Med14 S983A cells. See also *SI Appendix*, Fig. S5 and Datasets S9–S12.

To further explore the role of Med14 phosphorylation in Ex-4-driven beta cell transcription, we compared RNA-seq datasets of WT and Med14 S983A INS-1 cells treated with Ex-4 for 1, 8, or 16 h. The transcriptional response to sustained (16 h) Ex-4 exposure was significantly blunted in Med14 S983A mutant cells (Fig. 5*A* and Datasets S9–S11); but global transcription was relatively unaffected in Med14 S983A mutants (*SI Appendix*, Fig. S4*A*), suggesting that MED14 acts on a subset of genes that function in ER stress, metabolism, and insulin secretion (Fig. 5*B*). Cluster analysis of genes uniquely induced in WT cells after 16 h Ex-4 treatment (n = 636; FC > 1.5, padj. < 0.05) revealed that S983A mutation of Med14 reduces the response to Ex-4 in part by increasing the basal expression of these genes (*SI Appendix*, Fig. S4*B*, clusters 1 and 2). Consistent with this finding, KEGG analysis revealed significant overlap in pathway enrichment between genes induced upon prolonged Ex-4 exposure and genes up-regulated in Med14 S983A mutants under basal conditions (Fig. 1*C* and *SI Appendix*, Fig. S4 *C* and *D*).

Beta cells respond to increases in circulating glucose concentrations by inducing glycolytic and lipogenic gene expression. However, chronic overexpression of glycolytic and lipogenic programs is also associated with loss of metabolism-secretion coupling, beta cell failure, and diabetes, indicating that appropriate regulation of metabolic genes is crucial for beta cell function. Having seen enrichment of metabolic genes after sustained Ex-4 treatment in standard RPMI media (11 mM glucose), we next evaluated the role of Med14 S983 in regulation of glycolytic gene expression. Glycolytic gene induction by Ex-4 is mostly suppressed in Med14 S983A mutant cells. In addition, genes involved in cyclic pyruvate pathways, cytoplasmic malate dehydrogenase 1 (*Mdh1*) and the mitochondrial citrate carrier (*Slc25a1*), are strongly induced by Ex-4 in WT, but not mutant cells. (Fig. 5*C*). Moreover, pyruvate dehydrogenase kinase genes *Pdk1* and *Pdk2* are down-regulated in mutant cells, indicating increased entry of pyruvate into the TCA cycle and several TCA cycle genes are induced as well (*Sucla2*, *Idh*, *Sdhc*, *Ogdh*) (*SI Appendix*, Fig. S4*E*). These observations suggest that Ex-4 can accelerate metabolic pathways that are linked to insulin secretion. To test this idea, we measured oxygen consumption rate (OCR) in WT and mutant cells exposed to FSK for 16 h. Treating WT cells with FSK induced OCR under low (5 mM) and high (20 mM) glucose conditions. In contrast, Med14 S983A cells had significantly reduced OCR and were not responsive to FSK or high glucose exposure (Fig. 5*D*). Together, these results suggest that Med14 phosphorylation supports accelerated glucose metabolism and ATP-linked respiration upon Ex-4 exposure.

Lipogenic and cholesterol synthesis genes were induced in WT INS-1 cells after prolonged Ex-4 treatment in WT cells (Fig. 5 *C*, *Middle* and *Bottom*). Basal expression of these genes was mostly increased in Med14 S983A mutants, while activation after 16 h Ex- 4 was blunted, indicating that Med14 S983A modulates lipogenic genes in both basal and stimulated states (Fig. 5 *C*, *Middle* and *Bottom*). Western blot analysis revealed that Ex-4 treatment resulted in increased SREBP-1 cleavage in WT but not Med14 S983A mutant cells. In keeping with increased basal cholesterol synthesis gene expression, full-length and processed SREBP-1 were substantially increased in Med14 S983A mutant cells versus WT (Fig. 5*E*). However, these increases in activated SREBP-1 levels were not sufficient for induction of SREBP target gene protein levels in mutant cells, consistent with earlier work showing that differences in metabolic gene expression are often more pronounced at the protein rather than the mRNA level (*Ldlr*; Fig. 5*E*) (37). Nutrient-induced SREBP target gene expression was disrupted in Med14 S983A mutant cells as well. Indeed, expression of *Ldlr* and *Hmgcs1* was significantly lower in lipid starved Med14 S983A mutant cells compared to WT, while repression by cholesterol treatment was blunted, resulting in higher SREBP target gene expression in nonstarved conditions. Together, these data indicate Med14 S983 plays an important role in promoting lipid homeostasis in part by enabling SREBP target gene regulation.

We noticed that increased cleavage of SREBP -1 in mutant cells coincided with induction of acyl-CoA synthases (*Acss*, *Acsl*) and *Fasn*, and suppression of genes involved in fatty acid and branched chain amino degradation (*Cpt1a*, *Acadsb*, *Cyp4a8*) (Fig. 5 *C*, *Bottom* and *E*). These results suggest an imbalance in intracellular energy stores in mutant cells. To test this possibility, we compared lipid profiles of WT and Med14 S983A mutant cells by untargeted lipidomics screening. Surprisingly, storage lipids (triglycerides, free fatty acids and cholesterol esters) were mostly depleted in mutant cells, while phospholipids and diacylglycerol were generally increased (Fig. 5*G* and Dataset S12). Together, our data indicate that mutation of Med14 S983 impairs inhibitory feedback signaling and storage lipid accumulation, resulting in increased basal expression of Ex-4-induced genes. Mutation analysis of the Med14 ortholog in *S. cerevisiae* (*RGR1*) revealed that deletion of the *RGR1* C-terminal region results in depletion of reserve carbohydrate stores and elevated cAMP levels, suggesting that the role of Med14 in nutrient storage balance and cAMP signaling is conserved in eukaryotes (38).

### Med14 S983 Controls Gene Activation by Ex-4 in Primary Mouse Islets.

**Fig. 6.**
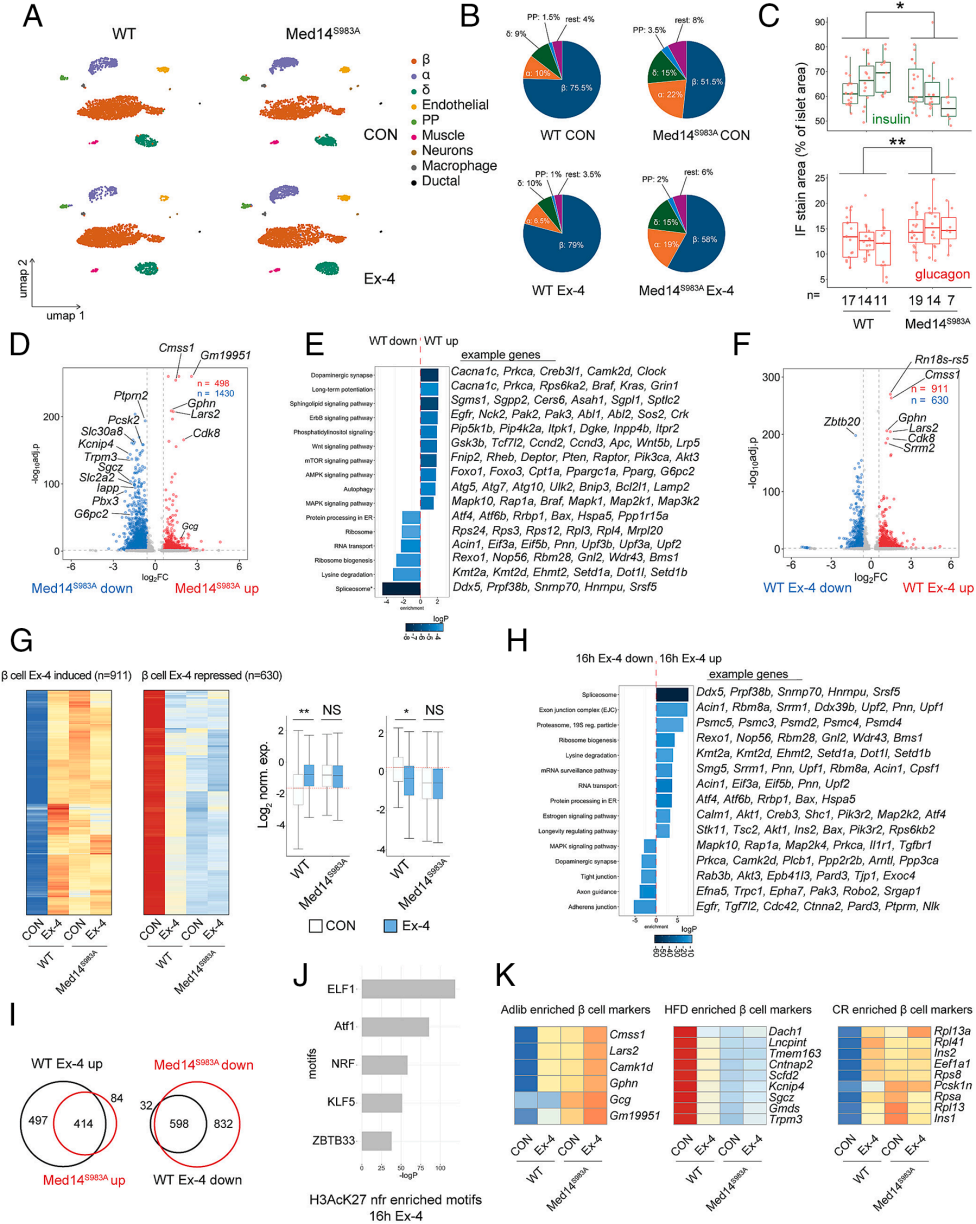
Med14S983 controls beta cell plasticity and gene activation by Ex-4 in primary mouse islet tissue. (*A*). Uniform manifold approximation and projection (UMAP) representation of WT and Med14 S983A mutant pancreatic islets untreated (*Top*) or treated for 16 h with 10 nM Ex-4 (*Bottom*). 2,500 nuclei are shown per sample. Cell Type annotations are indicated. (*B*). Relative cell type composition per condition. (*C*). Percentage of insulin (*Top*) and glucagon (*Bottom*) positive stain area per islet in WT and Med14 S983A mutant pancreatic sections (three animals for each condition). The number of islets measured per animal is indicated. Significance was determined with the Mann–Whitney *U* test. *P < 0.04, **P < 0.02. (*D*). Volcano plot comparing pseudobulk gene expression in WT and Med14 S983A mutant β cells. Relevant differentially expressed genes (FC >= 1.5; P Adj. <= 0.05) are highlighted. (*E*). KEGG pathway enrichment of differential genes in WT and Med14 S983A mutant β cells. Example genes for each pathway are shown on the *Right*. (*F*). Volcano plot comparing pseudobulk gene expression by Ex-4 (10 nM) in β cells (16 h). Relevant differentially expressed genes (FC >= 1.5; P Adj. <= 0.05) are highlighted. (*G*). Heatmap of genes induced (*Left*) and repressed (*Middle*) by Ex-4. Relative expression in WT and Med14 S983A mutant beta cells is shown. Boxplots showing Log2 expression values of signal shown in the heatmaps. *P < 0.0001 by one-way ANOVA with Holm-Sidak post hoc test. (*H*). KEGG pathway enrichment of genes regulated by Ex-4 (10 nM) in WT β cells (16 h). Example genes for each pathway are shown on the *Right*. (*I*). Overlap between genes significantly induced in Med14 S983A mutant cells (WT CON/Med14 S983A CON: FC <= 1.5; P Adj. <= 0.05) and genes significantly induced by Ex-4 in WT cells (16 h) (WT Ex-4/WT CON: FC >= 1.5; P Adj. <= 0.05) (*Left*). Overlap between genes significantly repressed in Med14 S983A mutant cells (WT CON/Med14 S983A CON: FC >= 1.5; P Adj. <= 0.05) and genes significantly repressed by Ex-4 in WT cells (16 h) (WT Ex-4/WT CON: FC <= 1.5; P Adj. <= 0.05) (*Right*). (*J*). Enrichment of TF binding motifs in nucleosome-free regions (nfr) of H3K27Ac decorated enhancers annotated to beta cell genes induced by Ex-4 (16 h). H3AcK27 ChIP data from whole primary mouse islet tissue (GEO: GSM3604411). (*K*). Heatmap depicting expression of marker genes of beta cell subclass enriched in adlib fed (*Left*), high fat diet (HFD) fed (*Middle*) and caloric restricted (CR) (*Right*) beta cells (41). See also *SI Appendix*, Fig. S6 and Datasets S13–S15.

To determine the role of Med14 S983 in pancreatic islets, we undertook single nucleus RNA-seq (snRNA-seq) of cultured islets isolated from WT and whole-body knock-in Med14 S983A mutant mice we generated for this study. We identified alpha, beta, delta, and PP cell populations in WT and mutant tissue (Fig. 6*A*). Relative beta cell numbers were significantly reduced while alpha and delta cell content increased in Med14S983A mutant islets, similar to the shift in alpha-to-beta cell ratio observed in Type 2 diabetes (Fig. 6*B*) (39, 40). Immunofluorescence analysis confirmed a decrease in insulin and an increase in glucagon positive staining area per islet in Med14 S983A mutant islets (Fig. 6*C* and *SI Appendix*, Fig. S6*A*). Pseudobulk analysis of gene expression in beta cells revealed repression of several cell type–specific genes in mutant beta cells (e.g., *Iapp*, *Slc2a2*, *Slc30a8*), while glucagon (*Gcg*) gene expression was up-regulated in mutant beta cells (Fig. 6*C* and Dataset S13). KEGG pathway analysis revealed repression of pathways involved in growth, differentiation, secretion, and cell adhesion (*mTOR, MAPK, Rap1, Wnt, EGFR*) in Med14 S983A mutants, suggesting that impaired expression of signaling molecules underlies diminished growth and differentiation in mutant beta cells (Fig. 6*D*). Consistent with our observations in INS-1 cells, negative modulators of cAMP signaling (*Grk5, Pde3b*) were repressed in Med14S983A mutant islet cells, confirming that Med14 is an effector of the cAMP pathway in primary cells (*SI Appendix*, Fig. S6*A*). Furthermore, mediator subunits *Cdk8*, *Med12,* and *Med25*, activating histone H3 methyl transferases (*Kmt2a, Ehmt2, Setd1a, Dot1l*) as well as genes controlling posttranscriptional processing of mRNA (splicing, mRNA transport and surveillance, exon junction complex) are induced in Med14 S983A mutant cells, while the conserved noncoding RNA 7SK, a component of the repressor to the transcription elongation complex P-TEFb, is repressed in mutant cells (Fig. 6 *C* and *D*). Overall, these findings indicate that mutation of Med14S983 triggers feedback mechanisms that compensate for decreased mediator activity and elongation-dependent gene transcription downstream of cAMP signaling.

Exposure of cultured islets with Ex-4 for 16 h resulted in a broad transcriptional response in WT pancreatic beta cells (n = 911 Ex-4/CON >= 1.5, padj. <= 0.05/n = 630 Ex-4/CON <= 1.5, padj. <= 0.05) (Fig. 6*E* and Dataset S14). Consistent with a role of Med14 S983 in Ex-4 mediated transcription, Med14 S983A mutant beta cells were not responsive to Ex-4 treatment (Fig. 6*F* and Dataset S15). In agreement with our observations in INS-1 cells, the basal gene expression profile of primary Med14 S983A mutant beta cells mirrors that of Ex-4-stimulated WT cells. Indeed, ~83% (414/498) of genes induced in Med14 S983A mutant cells (basal) compared to WT are also induced by Ex-4 in WT cells, while ~41% (598/1430) of genes repressed in Med14 S983A mutant cells compared to WT are also repressed by Ex-4 in WT cells (Fig. 6 *G* and *H*). Motif enrichment analysis of NFRs in H3K27Ac active enhancers of mouse islets revealed enrichment of Atf1/CREB motif in enhancers annotated to Ex-4 induced genes (Fig. 6*I*), pointing to a role of CREB in Ex-4 induced gene expression in beta cells.

Recent work linked long-term feeding regimens (adlib, caloric restriction (CR), high fat (HF)) to a shift in beta cell transcriptional states in primary islets using single cell RNA-seq (41). Remarkably, sustained exposure of Ex-4 induced beta cell marker genes that are enriched in CR (e.g., *Rpl13a*, *Rpl41*, Ins1, *Ins2*, *Eef1a*) and adlib (e.g., *Cmss1*, *Lars2*, *Camk1*) mice and depleted marker genes enriched in and HF (e.g., *Dach1*, *Lncpint*, *Tmem163*, *Cntnap2*) mice in a Med14-dependent manner (Fig. 6 *C*, *E*, and *J*). These findings indicate that sustained Ex-4 shifts beta cells to an active metabolic state that favors beta cell survival (41), while confirming a role of Med14 S983 in regulating insulin gene induction by Ex-4 in primary beta cells (Fig. 6*J*).

Finally, we tested the role of Med14 S983 in transcriptional response to FSK (16 h) in primary islets. The role of Med14S983 in FSK induced gene regulation appears more limited in primary beta cells (*SI Appendix*, Fig. S6*B* and Datasets S16 and S17). However, genes induced by 16 h exposure to FSK in a Med14 S983-dependent manner drive relevant processes like vesicle trafficking (*Astn2, Hid1, Rab20*), actin rearrangement (*Bcar1, Peak1, Ctnnal1*), calcium mobilization (*Ryr3*), protein folding in the ER (*Pdia6*), growth (*Shc4*), and PKA activity (*Crybg3)* in beta, but not alpha, delta, or PP cells (*SI Appendix*, Fig. S6*C*). Furthermore, a number of genes in this set (*Peak1*, *Astn2*, *Bcar1, Ctnnal1, Ppfibp2*) is associated with significant diabetes-related risk loci (*SI Appendix*, Fig. S6*D*) (42). ChIP-seq analysis in primary mouse islets confirmed CREB binding in active enhancers in Med14 S983-dependent diabetes-associated loci *Bcar1/Ctrb1* and *Peak1/Hmg20a* (*SI Appendix*, Fig. S6*E*). Previous work showed that the common SNP rs7202877 in the *Bcar1/Ctrb1* locus significantly associates with decreased GLP-1 induced insulin secretion (43). Further inspection of the *Bcar1/Ctrb1* locus in INS-1 cells revealed a CREB bound inducible enhancer that was inhibited in Med14 S983A mutant cells, directly confirming the role of Med14 S983 in promoting FSK-induced gene induction in the *Bcar1/Ctrb1* locus (*SI Appendix*, Fig. S6 *F* and *G*). These observations are consistent with the identification of rs7202877 as an expression quantitative trait locus (eQTL) controlling expression of *Ctrb1,2* in humans (43). Given the abundance of disease-associated SNPs genomic regions that regulate gene expression in human islets (44–46), these findings link Med14 S983 phosphorylation with sustained Ex-4 treatment in the induction of beta cell-specific transcripts with crucial roles in maintaining glucose homeostasis.

## Discussion

Initially identified as an insulin secretagogue (47, 48), GLP-1 and its stable mimetics were found to amplify the beta cell response to high glucose levels in a sustained fashion. Indeed, GLP-1 induces beta cell adaptation to increased fuel pressure by promoting differentiation, suppressing autophagy, lowering ER stress and rewiring metabolism (49). These chronic responses were long thought to be transcriptionally driven (1, 2, 10, 12, 50) with CREB being one of the most universally observed mediators of GLP-1 action. Triggering of the GLP-1 receptor in beta cells results in induction of the second messenger cAMP, but how this ubiquitous signaling pathway regulates cellular gene expression in specific cellular contexts is poorly understood.

We found that sustained Ex-4 exposure elicits a broad transcriptional response in INS-1 and primary beta cells that includes induction of genes involved in metabolism, actin remodeling, differentiation, and growth factor signaling. We confirmed that this gene response is driven by cAMP, as it is largely mimicked by exposure of the adenylyl cyclase agonist forskolin. In exploring the mechanistic underpinning of the Ex-4 response, we identified the essential mediator scaffolding subunit Med14 as an in vivo PKA target, which undergoes Ser983 phosphorylation in response to Ex-4. Upon phosphorylation, Med14 promotes cAMP-induced gene expression, particularly cell-specific delayed-early gene targets.

Med14 S983A mutation also impairs regulation of SREBP target gene expression both by Ex-4 and cholesterol, showing that a single residue on mediator controls lipid homeostasis through both nutrient and hormonal inputs. Given the central role of cAMP-PKA signaling in lipid metabolism, future studies should illuminate the role of Med14 phosphorylation on lipogenesis in other metabolic tissues.

Our single nucleus RNA-seq data in primary mouse islets confirmed a broad transcriptional response after prolonged Ex-4 exposure in primary beta cells. In addition, we observed a shift in endocrine cell composition of Med14 S983A knock-in mutant primary islets, with alpha cells being enriched in Med14 S983A compared to WT islets, resembling endocrine cell ratios in islets of Type 2 diabetic mice. Med14 S983A mutant beta cells are also depleted in marker genes recently identified in a beta cell subclass that expands upon high fat diet feeding, suggesting Med14 phosphorylation may play a role in the adaptive response to nutrient status. Together, these data suggest a pivotal role of Med14 phosphorylation in GLP-1-induced beta cell differentiation and adaptation to increased fuel pressure. However, our mechanistic studies were conducted in rodent models and further work in primary human islet tissue should reveal if our findings are relevant to GLP-1 activity in the context of human metabolism and diabetes.

Med14 S983 is contained within a 200 amino acid Intrinsically disordered Region IDR. IDRs have been shown to promote the formation of transcriptionally hyperactive condensates containing RNA Pol II and coactivators that selectively drive expression of cell-restricted genes by activating distal enhancers. Mounting evidence supports a role for amino acid and RNA charge balance in promoting compartmentalization and transcriptional activity (51–53). Specifically, localized enrichment of charged residues in Med1 IDRs was found critical for partitioning of activators inside condensates (54). Phosphorylation-induced negative charge inside IDRs could alter the size, composition, and function of molecular condensates in response to hormonal stimuli. Although Ser983 phosphorylation does not appear to change the subunit composition of Mediator, Ser983 phosphorylation of Med14 could trigger structural changes in Mediator that compromise its ability to recruit the transcriptional machinery to relevant enhancers. Altogether, our studies unexpectedly reveal that phosphorylation of a single serine residue in the Med14 protein plays a significant role in the response to GLP-1 agonists and more broadly in the metabolic response to hormones.

## Materials and Methods

### INS-1 Cell Culture.

INS-1 rat insulinoma cells were grown at 37C with 5% CO_2_ in RPMI-1640 media (Corning) supplemented with 10% FBS (Gemini), 2 mM glutamine (Mediatech), 1 mM sodium pyruvate (Mediatech), 100 μg/mL penicillin-streptomycin (Mediatech), and 0.05 mM β-mercaptoethanol.

### Western Blotting.

Western blotting was performed using a standard procedure. Protein extract was run on polyacrylamide gels at 30 mA per gel and transferred onto Nitrocellulose membranes at 300 mA. Membranes were washed with TBS 0.1% Tween-20 (TBST) and exposed to antibody in TBST with 5% milk overnight at 4C. Membranes were then washed three times in TBST for 15 min. each and incubated for 1 h with HRP coupled secondary antibody (1/5,000 dilution) followed by three more TBST washes. After 10 s exposure to SuperSignal West Pico PLUS Chemiluminescent Substrate (Thermo Scientific 34578), blots were exposed to imager film and imaged with an X-ray film processor.

### Quantitative PCR.

RNA was extracted from cultured cells or tissue with Trizol (Invitrogen 15596026)/chloroform. cDNA was synthesized from 1 μg total RNA using the Transcriptor First Strand cDNA Synthesis Kit (Roche 04897030001) according to the manufacturer’s instructions. Quantitative PCR was performed using LightCycler 480 SYBR Green I Master mix (Roche 04887352001) in a LightCycler 480 II (Roche).

### RNA-seq.

Phenol-chloroform extracted total RNA was cleaned up using the Direct-zol RNA MiniPrep kit (Zymo Research). 1 ug total RNA was used to build RNA-seq libraries with NEBNext Ultra II Directional RNA Library Prep Kit for Illumina (NEB E7760). 2 μL of 1:100 diluted ERCC Spike-In Mix 1 (Invitrogen 4456740) was added to total RNA. Paired-end sequencing of libraries was performed on the NovaSeq X Plus sequencer (Illumina).

### Chromatin Immunoprecipitation (ChIP).

ChIP was performed as described earlier. Briefly, cells were fixed in 0.75% formaldehyde for 10 min and quenched with 125 mM glycine for 5 min. Cells were washed and scraped in ice-cold PBS. Cell pellets were resuspended in buffer LB3 (10 mM Tris-HCl pH 8.0, 100 mM NaCl, 1 mM EDTA, 0.5 mM EGTA, 0.1% Na-deoxycholate, 0.5% N-laurylsarcosine, protease inhibitor cocktail, Sigma P9599) and sonicated (Active Motif EpiShear Probe Sonicator). 35 μL protein A agarose beads (Thermo Scientific 20333) were washed twice with PBS + 0.1% BSA and coupled with antibody for 4 h in PBS + 0.1% BSA at 4 °C with rotation. For ChIP, 500 μL extract was added to antibody coupled beads and incubated overnight at 4 °C with rotation.

Beads were washed three times in 500 μL wash buffer 1 (20 mM Tris-HCl pH 7.4, 150 mM NaCl, 2 mM EDTA, 0.1% SDS, 1% Triton X-100) and three times in wash buffer 2 (20 mM Tris-HCl pH 7.4, 250 mM LiCl, 1 mM EDTA, 1% Triton X-100, 0.7% Na-deoxycholate). Elution was then performed by incubating beads in 50 μL elution buffer 1 (TE, 1% SDS) for 15 min at 50 °C and 50 μL elution buffer 2 (TE, 1% SDS, 300 mM NaCl) for 15 min at 50 °C while shaking. Both elutions were combined and incubated with proteinase K and RNase A for 2 h at 37 °C. DNA–protein complexes were decrosslinked at 65 °C while shaking overnight. ChIP DNA was purified using Agencourt AMPure XP beads (Beckman Coulter A63881). 10 ng ChIP DNA was used to build libraries using the NEBNext Ultra II DNA library Prep Kit for Illumina (NEB E7645) according to the manufacturer’s instructions. ChIPseq reads were aligned to the rn6 reference genome and analyzed for peak detection, read quantification, and motif enrichment using HOMER (55). For detection of nucleosome-free regions in active promoters/enhancers, the following Homer command was used: findPeaks H3K27Ac_tagdirectory -o out -i input_tagdirectory -size 1000 -minDist 2500 -nfr.

### ATAC-seq.

ATAC-seq was performed as described previously (56), with minor modifications. Briefly, transposition was performed on 50,000 nuclei with Tagment DNA enzyme and buffer for 30 min at 37C according to the manufacturer’s instructions. DNA was isolated using the Qiagen MinElute Reaction Cleanup Kit and amplified with Illumina/Nextera i5 common and i7 index adapters.

ATAC-seq libraries were sequenced on a NovaSeq X (Illumina) (~2.5E8 reads per library). Reads were trimmed with Trim Galore, aligned to the rn6 reference genome using bwa mem. Mitochondrial, low quality mapped reads and duplicated reads were filtered out with samtools and peaks were called with macs2. Processed fragments were then shifted and sorted into nucleosome-free, mono-, di-, and trinucleosome spanning regions with ATACseqQC (57). Footprinting analysis was performed on short (nucleosome-free) fragments using TOBIAS (58).

### Pancreatic Islet Isolation.

Anesthetized animals were killed by cervical dislocation. Collagenase buffer [HBSS, 2 mM CaCl2, 20 mM HEPES pH 7.4, Collagenase P (0.3 mg/mL)] was injected in the common bile duct and pancreas was digested in a water bath at 37C for 14 min. Digestion was stopped on ice and by addition of 15 ml ice-cold HBSS. Pancreases were disrupted by vigorous shaking and filtered through a 500 μm mesh. Next, digested tissue was settled by gravity on ice for 10 min. Supernatant was aspirated and tissue washed with HBSS + 10% FBS. Tubes were centrifuged at 220x g for 2 min and dried pellets were resuspended in 10 ml Histopaque. Cell suspension was overlaid with 10 ml HBSS and centrifuged at 850x g for 15 min with brake off. Histopaque containing islets was collected, washed with HBSS + 10% FBS, centrifuged at 220x g for 2 min. Islet pellets were resuspended in 3 ml RPMI + 10% FBS and hand picked three times under a microscope. Islets were cultured overnight and hand picked again before Ex-4 treatment and harvesting.

### Nuclear Isolation of Pancreatic Islets for snRNA-seq.

Frozen islets (~100 per sample) were resuspended in 10 ml of lysis buffer (0.32 M sucrose, 5 mM CaCl2, 3 mM Mg-Acetate, 0.1 mM EDTA pH 8, 10 mM Tris-HCl pH 8, 1 mM DTT, 0.1 % Triton X-100 in DEPC water) supplemented with 10 μL protease inhibitor and 7.5 μL RNase inhibitor (Promega N251B). Suspensions were homogenized on ice in a 15 ml Dounce homogenizer with 15 strokes of the loose (A) and 35 strokes of tight (B) pestle. Nuclei were centrifuged for 5 min at 500 g at 4C without brake. Nuclear pellet was resuspended in 400 μL sort buffer (2% BSA, 1 mM EDTA pH 8, 5 μL RNase inhibitor, 1 μg/mL DAPI in dPBS) and filtered through a 30 um CellTric filter into a FACS tube. DAPI-positive nuclei were gated first, followed by exclusion of debris using forward and side scatter pulse area parameters (FSC-A and SSC-A), exclusion of aggregates using pulse width (FSC-W and SSC-W). A BD Influx sorter was used to isolate nuclei, with PBS for sheath fluid (100-μm nozzle was used for cells with sheath pressure set to 20PSI). The nuclei were sorted directly into 1.5 mL eppendorf tubes containing 200 μL collection buffer (5% BSA in dPBS, 200U RNase inhibitor) using the “1-drop Pure” sort mode.

### snRNAseq Library Construction.

Isolated nuclei were fixed using Evercode Nuclei Fixation v3 (Parse Biosciences ECFN3300) according to the manufacturer’s instructions. Combinatorial barcoding of transcriptome on fixed nuclei transcriptome was performed using Evercode WT v3 (Parse Biosciences ECWT3300) according to the manufacturer’s instructions.

### Quantification and Statistical Analysis.

Statistical tests were performed with R or Excel. Details of the analysis used can be found in the figure legends. Error bars always indicate SD. Significance of differential gene expression in bulk RNA-seq was determined by DESeq2 on duplicates. Cutoffs for differential expression are indicated in the Results section. All experiments were performed in replicates with two independently generated Med14 S983A knock-in lines.

## Supplementary Material

Appendix 01 (PDF)

Dataset S01 (TXT)

Dataset S02 (TXT)

Dataset S03 (TXT)

Dataset S04 (TXT)

Dataset S05 (TXT)

Dataset S06 (TXT)

Dataset S07 (TXT)

Dataset S08 (TXT)

Dataset S09 (TXT)

Dataset S10 (TXT)

Dataset S11 (TXT)

Dataset S12 (XLSX)

Dataset S13 (CSV)

Dataset S14 (XLSX)

Dataset S15 (XLSX)

Dataset S16 (CSV)

Dataset S17 (CSV)

## Data Availability

Raw data can be accessed from GEO under the accession number GSE299280 (RNA-seq) (59), GSE299281 (ATAC-seq) (60), GSE299282 (snRNA-seq) (61) and GSE299399 (62). All other data are included in the article and/or supporting information.
