# Supplementary material for: Med14 phosphorylation shapes genomic response to GLP-1 agonists
Source: Proc Natl Acad Sci U S A. 2026 Mar 4;123(10):e2536772123. doi: 10.1073/pnas.2536772123 (PMC12974444; doi:10.1073/pnas.2536772123)
Supplement: Supplementary file 1 — Appendix 01 (PDF) [file pnas.2536772123.sapp.pdf]

PNAS

Supporting Information for

Med14 phosphorylation shapes genomic response to GLP-1 Agonist

Sam Van de Velde, Jungting Yu, K. Garrett Evensen, Edmund Pakhlevanyan, April E. Williams, Reuben J. Shaw, and Marc Montminy

Correspondence: Sam Van de Velde, Marc Montminy

Email: svandevelde@salk.edu

montminy@salk.edu

This PDF file includes:

Figures S1 to S5
Legends for Datasets S1 to S17
Extended Methods
Reagents and Resources
SI References

Other supporting materials for this manuscript include the following:

Description of Datasets S1 to S17

Figure S1

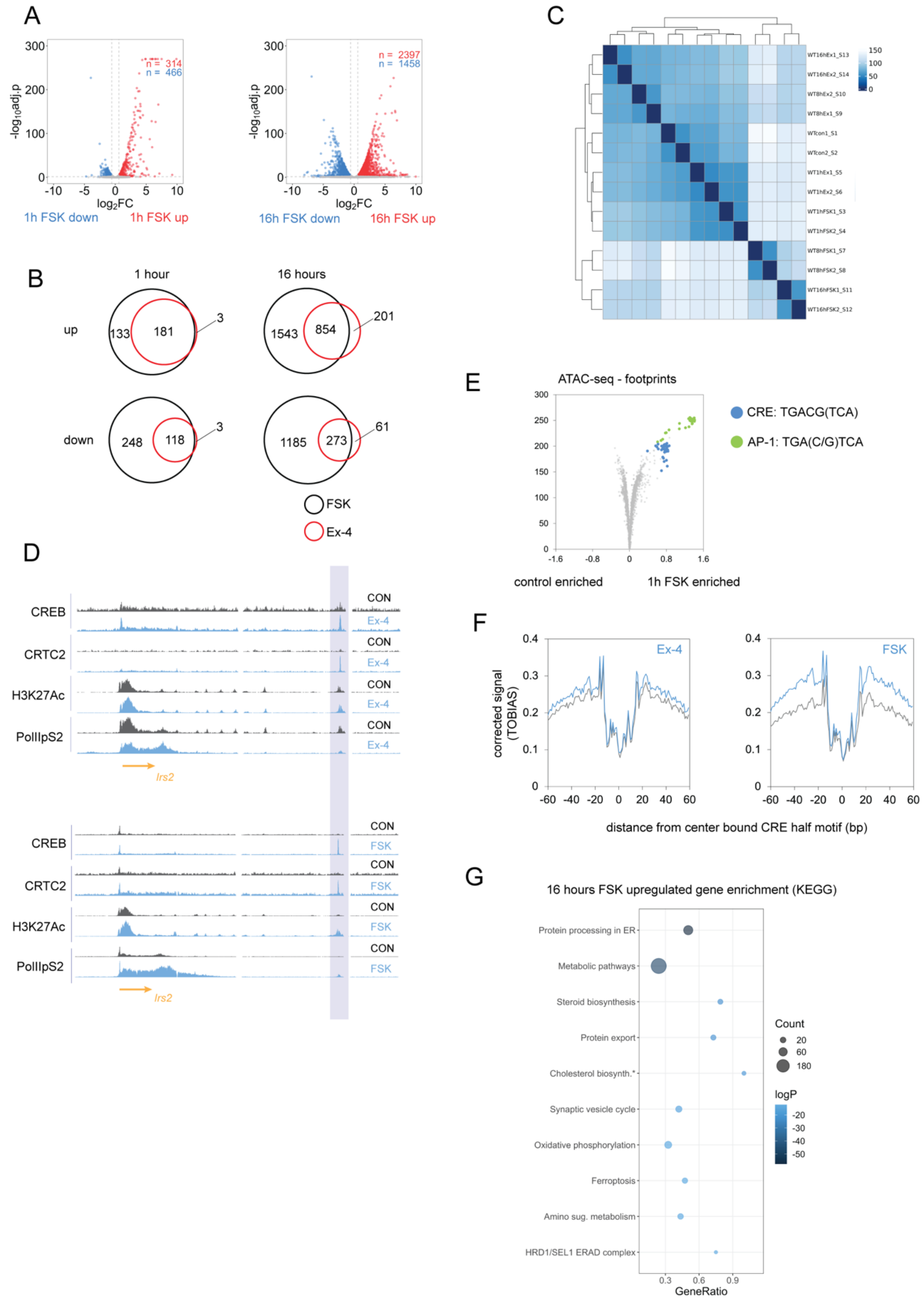
A
-log10adj.p
log2FC
n = 314
n = 466
1h FSK down
1h FSK up
n = 2397
n = 1458
16h FSK down
16h FSK up
B
1 hour
16 hours
up
down
133
181
3
1543
854
201
248
118
3
1185
273
61
FSK
Ex-4
C
D
CREB
CRTC2
H3K27Ac
PolIIpS2
CON
Ex-4
FSK
Irs2
E
ATAC-seq - footprints
CRE: TGACG(TCA)
AP-1: TGA(C/G)TCA
control enriched
1h FSK enriched
F
corrected signal (TOBIAS)
Ex-4
FSK
distance from center bound CRE half motif (bp)
G
16 hours FSK upregulated gene enrichment (KEGG)
Protein processing in ER
Metabolic pathways
Steroid biosynthesis
Protein export
Cholesterol biosynth.*
Synaptic vesicle cycle
Oxidative phosphorylation
Ferroptosis
Amino sug. metabolism
HRD1/SEL1 ERAD complex
Count
logP
GeneRatio

Figure S1: Overlap between Ex-4 and forskolin (FSK) transcriptional responses, related to Figure 1

(A) Differential gene expression after acute (1h, left) or sustained (16h, right) FSK exposure in INS-1 cells.
(B) Overlap of Ex-4 and FSK in induced (top) and repressed (bottom) genes after 1 hour (left) and 16 hours (right) exposure.
(C) Sample similarity across all conditions.
(D) Example ChIPseq tracks over the *Irs2* locus showing binding of CREB and CRTC induced by Ex-4 (top) and FSK (bottom) over an activated distal enhancer (shaded). Enhancer activity and CTD-phosphorylated RNA polymerase II are shown in H3AcK27 and Pol IIpS2 tracks, respectively.
(E) Volcano plot depicting change in footprint score over transcription factor binding motifs in accessible chromatin regions (ATAC) after 1 hour FSK treatment. Motifs corresponding to CREB response binding (CRE) and activator protein-1 (AP-1) are highlighted.
(F) ATACseq footprints over CRE half (CGTCA) sites after 1 hour Ex-4 (10 nM) (left) and FSK (10 μM) (right) treatment.
(G) KEGG pathway enrichment analysis of genes induced after 16 hours FSK (10 μM) exposure.

Figure S2

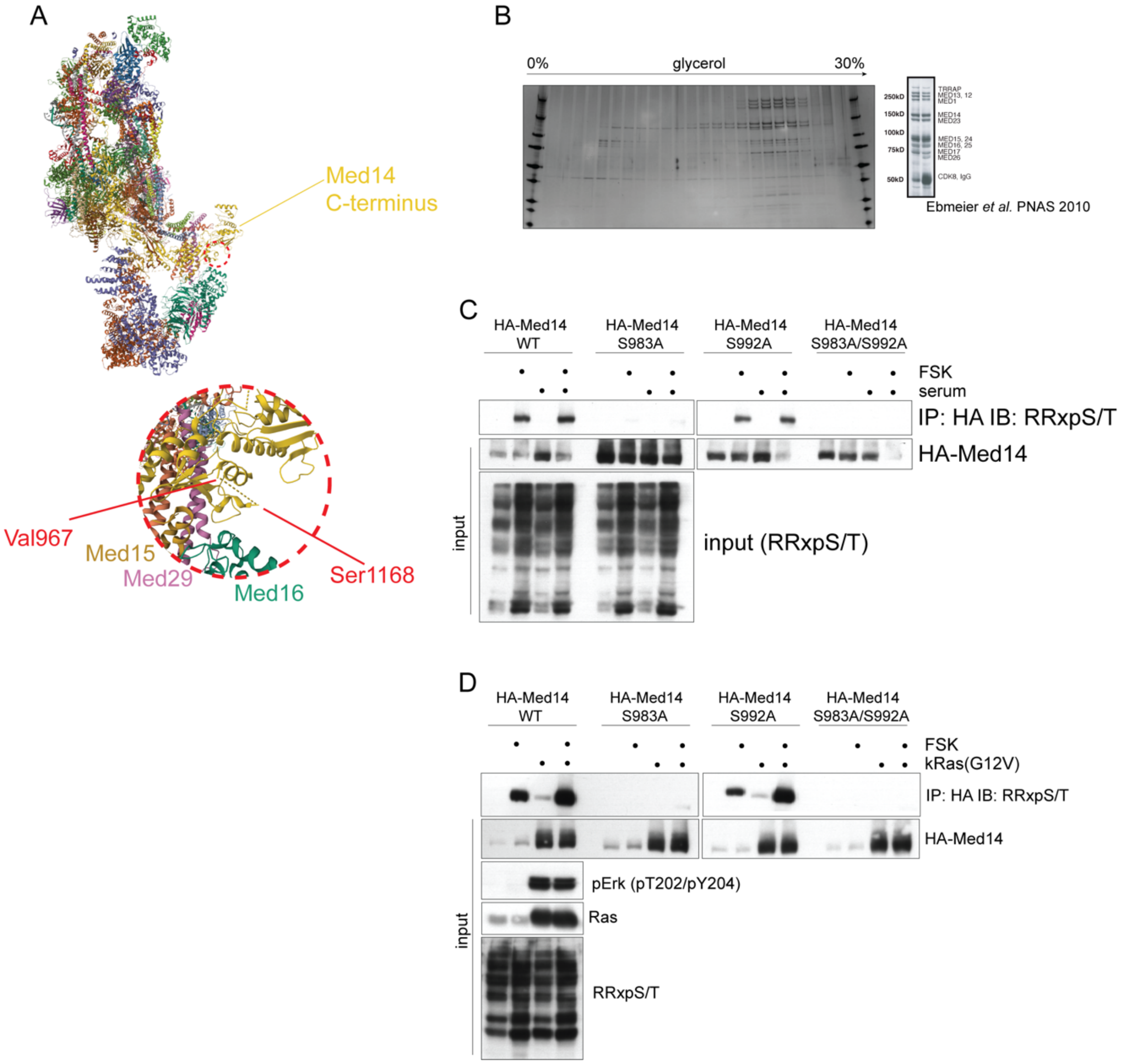

Figure S2: Med14 is a PKA target, related to Figure 2

(A) Structure of human mediator bound to pre-initiation complex (PDB 7LBM)[1]. Exposed Med14 CTD with unresolved IDR (Med14V967-S1168) is highlighted.

(B) Silver stain of purified mediator complex with Med14 S983A loaded on a 10%-30% glycerol gradient (left). Mediator silver stain reprinted form ref. [2] (right).

(C) Phosphorylation of Ser983 is not triggered by serum (10%) treatment and is independent of the Erk target site Ser992. 293T cells were serum starved for 4 hours prior to stimulation.

(D) Phosphorylation of Ser983 is not induced by expression of active Ras(G12V) mutant. Erk activation, Ras expression and PKA activity are shown in total extract.

Figure S3

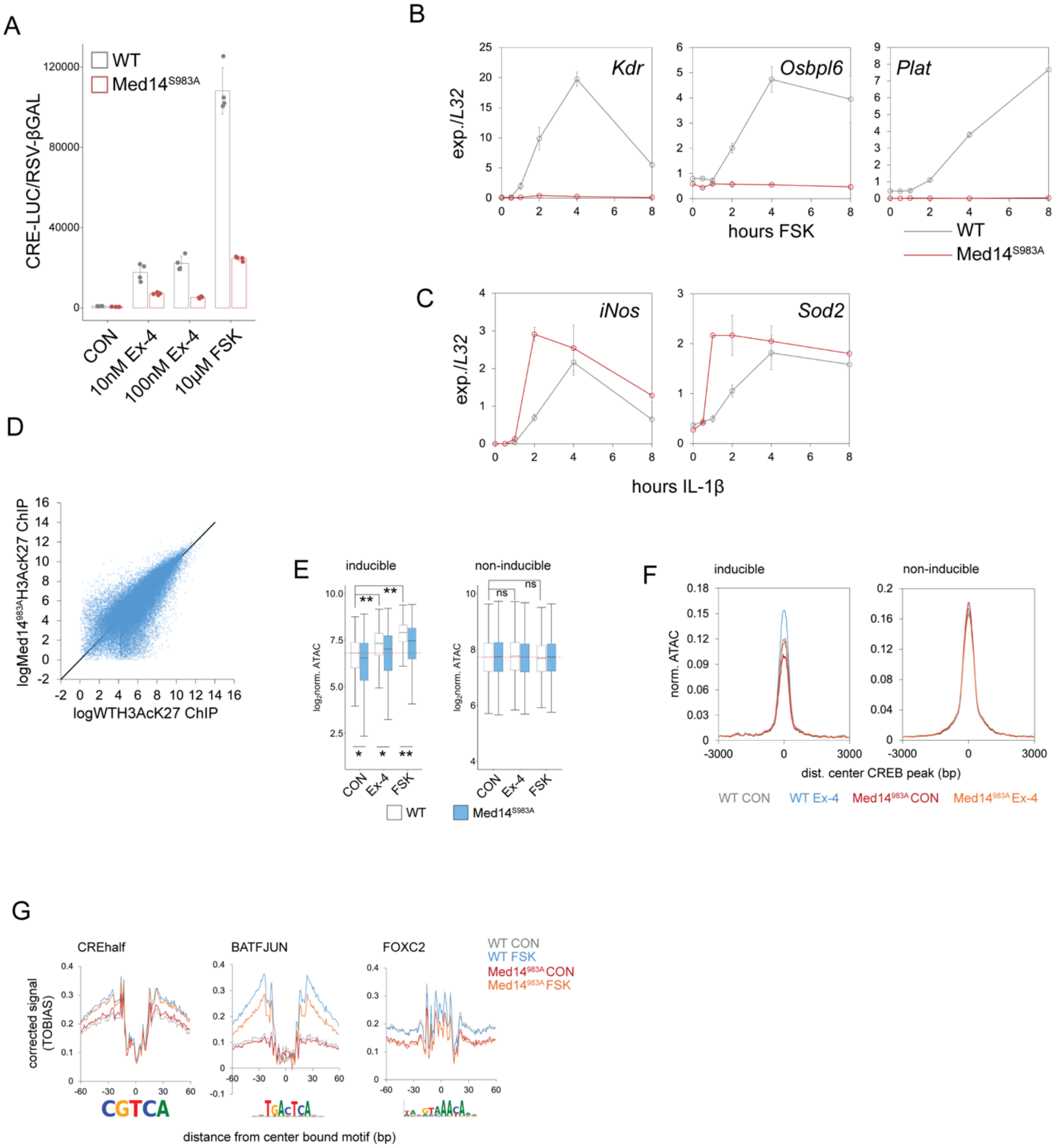
A
WT
Med14S983A
CRE-LUC/RSV-βGAL
120000
80000
40000
0
CON
10nM Ex-4
100nM Ex-4
10µM FSK
B
Kdr
Osbpl6
Plat
exp./L32
hours FSK
WT
Med14S983A
C
iNos
Sod2
exp./L32
hours IL-1β
D
logMed14983AH3AcK27 ChIP
logWTH3AcK27 ChIP
E
inducible
non-inducible
log2norm. ATAC
CON
Ex-4
FSK
WT
Med14S983A
F
inducible
non-inducible
norm. ATAC
dist. center CREB peak (bp)
WT CON
WT Ex-4
Med14983A CON
Med14983A Ex-4
G
CREhalf
BATFJUN
FOXC2
WT CON
WT FSK
Med14983A CON
Med14983A FSK
corrected signal (TOBIAS)
CGTCA
distance from center bound motif (bp)

Figure S3: Med14S983 phosphorylation promotes gene induction by activating enhancers, related to Figure 3

(A) Activation assay of WT and Med14 S983A cells transfected with a CREB-dependent (8xCRE) luciferase reporter. Cells were treated with Ex-4 or forskolin (FSK) as indicated for 6 hours. Signal was normalized by co-transfection of an RSV-βGAL construct.

(B) Time course Q-PCR for delayed-early (*Kdr*, *Osbpl6, Plat*) genes over 8 hours FSK (10 μM) treatment in WT and Med14 S983A mutant cells. Error bars show standard deviation.

(C) Time course Q-PCR for NF-κB target genes over 8 hours IL-1β (1 nM) treatment in WT and Med14 S983A mutant cells. Error bars show standard deviation.

(D) Scatter plot comparing H3AcK27 ChIP-seq reads in enhancers between WT and Med14 S983A mutant cells.

(E) Histogram of normalized ATAC-seq reads over inducible (left) and non-inducible (right) enhancers.

(F) Footprint profiles over example CRE (CREhalf), AP-1 (BATFJUN) and FOX (FOXC2) motifs in WT and Med14 S983 mutant cells. Cells were exposed to 10 μM FSK for 1 hour.

Figure S4

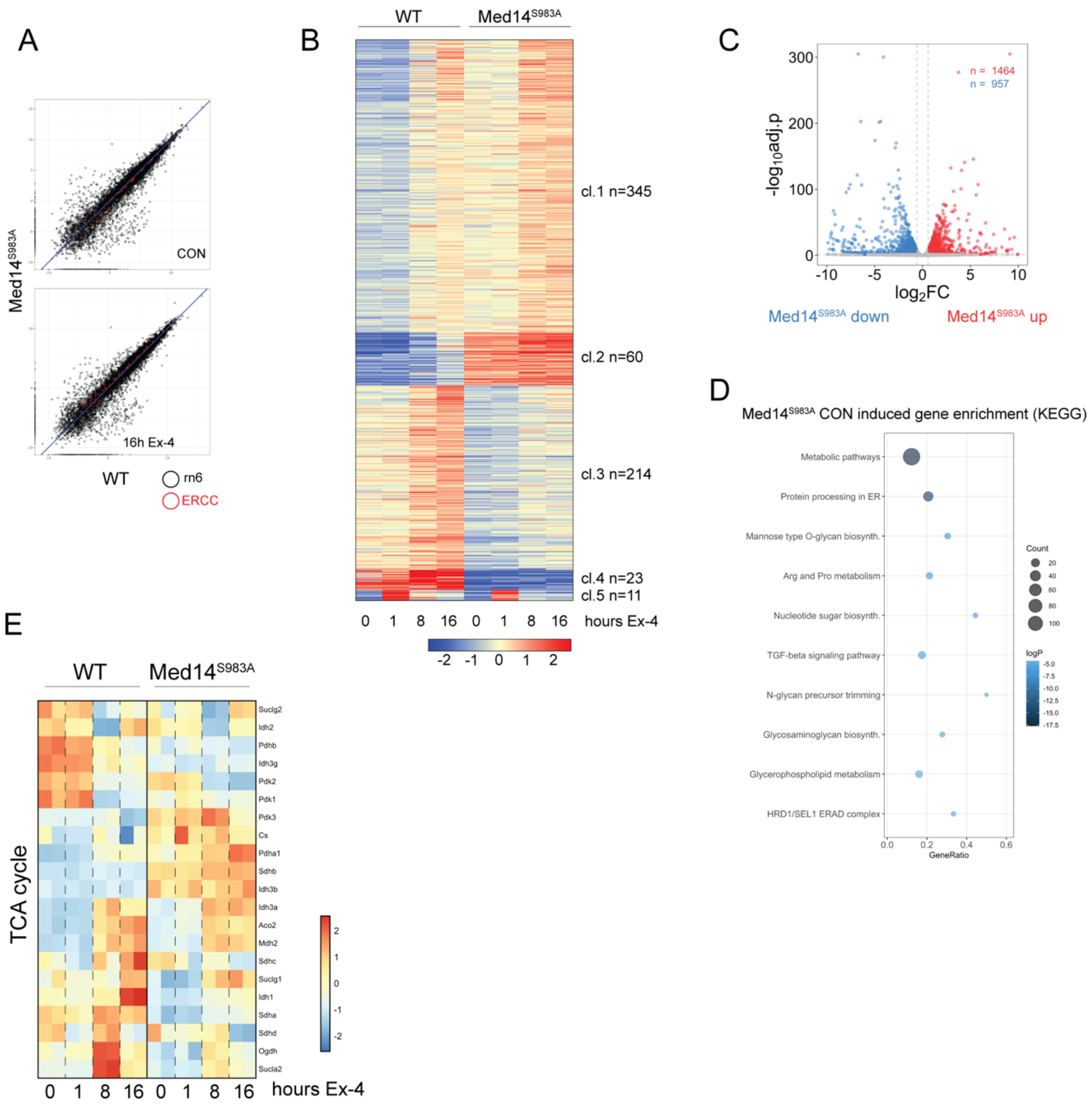

Figure S4: Ex-4 tunes metabolism through Med14, related to Figure 5

(A) Scatter plot comparing transcript counts between WT and Med14 S983A mutant cells in basal (top) and 16h Ex-4 (bottom) treated conditions. Spiked-in ERCC RNAs are highlighted in red.

(B) Clustered heatmap depicting expression of transcripts uniquely induced in WT cells (FC >1.5, Padj. < 0.05) after 16h Ex-4 exposure. Expression across indicated timepoints in WT and Med14 S983A mutant cells is shown.

(C) Volcano plot showing differential gene expression in WT and Med14 S983A mutant cells in basal conditions.

(D) KEGG enrichment of genes induced in Med14 S983A mutants in basal conditions.

(E) Heatmap showing expression of TCA cycle genes in WT and Med14 S983A mutant cells.

Figure S5

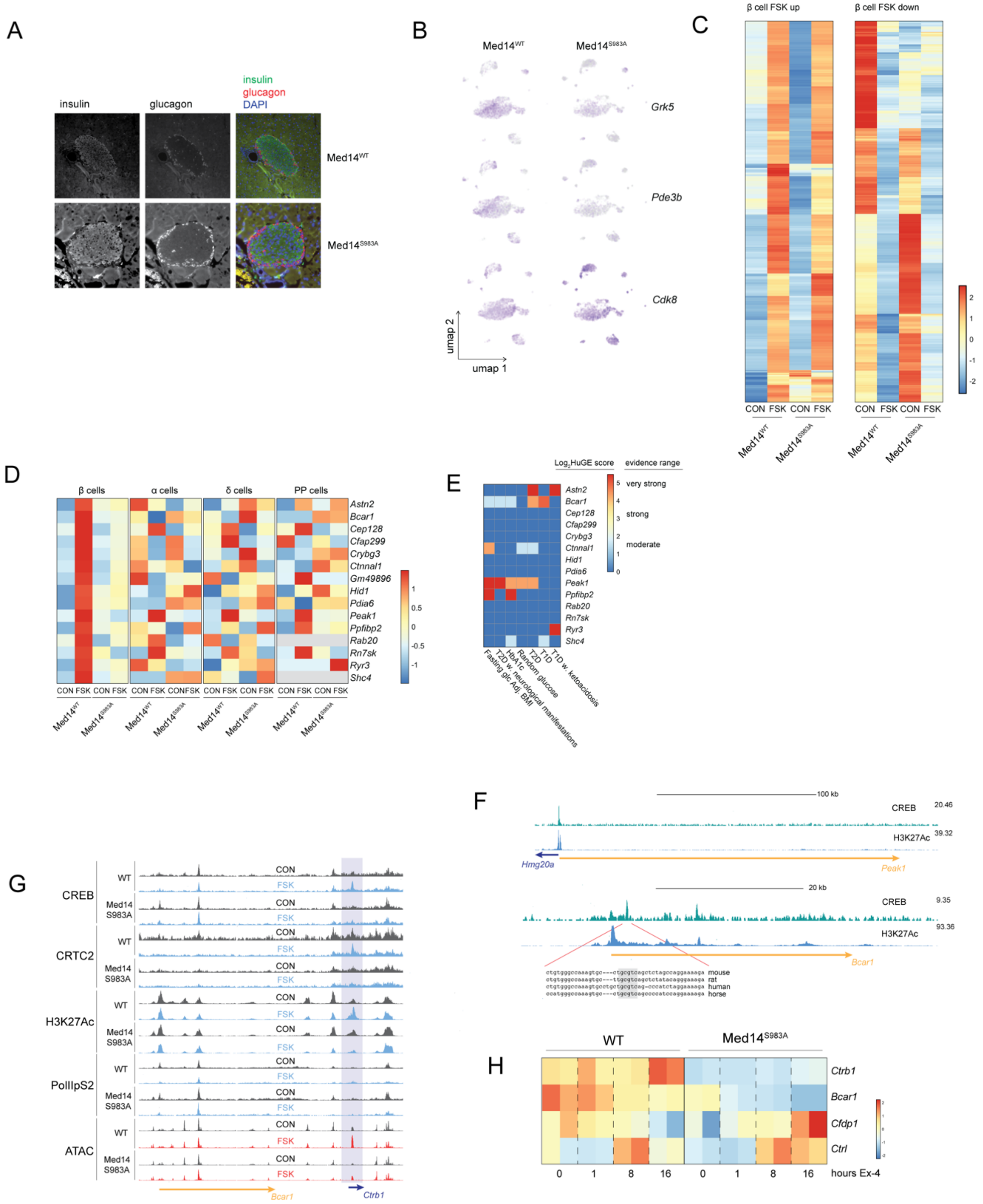
A
insulin
glucagon
insulin
glucagon
DAPI
Med14WT
Med14S983A
B
Med14WT
Med14S983A
Grk5
Pde3b
Cdk8
umap 2
umap 1
C
β cell FSK up
β cell FSK down
CON FSK CON FSK
Med14WT
Med14S983A
D
β cells
α cells
δ cells
PP cells
Astn2
Bcar1
Cep128
Cfap299
Crybg3
Ctnnal1
Gm49896
Hid1
Pdia6
Peak1
Ppfibp2
Rab20
Rn7sk
Ryr3
Shc4
E
Log2HuGE score
evidence range
very strong
strong
moderate
Fasting glc Adj. BMI
T2D w. neurological manifestations
HbA1c
Random glucose
T2D
T1D
T1D w. ketoacidosis
F
100 kb
CREB
H3K27Ac
Hmg20a
Peak1
20 kb
Bcar1
mouse
rat
human
horse
G
CREB
CRTC2
H3K27Ac
PolIIpS2
ATAC
WT
Med14 S983A
CON
FSK
Bcar1
Ctrb1
H
WT
Med14S983A
Ctrb1
Bcar1
Cfdp1
Ctrl
0 1 8 16 0 1 8 16
hours Ex-4

Figure S5: Med14S983 controls beta cell plasticity and gene activation by Ex-4 in primary mouse islet tissue, related to Figure 6

(A) Representative images of WT (top) and Med14 S983A (bottom) pancreatic islets stained for insulin and glucagon.
(B) UMAP plots highlighting expression of negative regulators of cAMP signaling (*Grk5*, *Pde3b*) and mediator kinase subunit *Cdk8*.
(C) Gene induction (left) and repression (right) by chronic FSK (16 hours) in WT and Med14 S983A mutant primary beta cells.
(D) Heatmap depicting induction of Med14 S983 controlled genes by FSK (16 hours) in WT and Med14 S983A mutants. β, α, δ and PP cells are compared.
(E) Heatmap showing Human Genetic Evidence (HuGE) scores [3] from hugeamp.org for genes shown in (Fig. 6F) across diabetes-related phenotypes.
(F) Chromatin occupancy of CREB and H3AcK27 in primary mouse islets across *Hmg20a/Peak1* and *Bcar1* genomic loci in primary mouse islets (GEO: GSM3604407, GSM3604411) [4]. Conserved intronic CREB response element in *Bcar1* intron is shown.
(G) ChIP-seq occupancy (top) and ATAC-seq (bottom) tracks over the *Bcar1/Ctrb1* locus in INS-1 cells. cAMP inducible enhancer is highlighted.
(H) Expression of genes in the *Bcar1/Ctrb1* locus in INS-1 cells after Ex-4 (10 nM) stimulation.

Datasets

Dataset S1. Differential Gene Expression WT CON v. 1h Ex-4, related to Figure 1
Dataset S2. Differential Gene Expression WT CON v. 8h Ex-4, related to Figure 1
Dataset S3. Differential Gene Expression WT CON v. 16h Ex-4, related to Figure 1
Dataset S4. Differential Gene Expression WT CON v. 1h FSK, related to Figure S1
Dataset S5. Differential Gene Expression WT CON v. 8h FSK, related to Figure S1
Dataset S6. Differential Gene Expression WT CON v. 16h FSK, related to Figure S1
Dataset S7. CREB bound cAMP Responsive Enhancers INS-1 BED rn6, related to Figure 3
Dataset S8. CREB bound non-responsive Enhancers INS-1 BED rn6, related to Figure 3
Dataset S9. Differential Gene Expression Med14 S983A CON v. 1h Ex-4, related to Figure 5
Dataset S10. Differential Gene Expression Med14 S983A CON v. 8h Ex-4, related to Figure 5
Dataset S11. Differential Gene Expression Med14 S983A CON v. 16h Ex-4, related to Figure 5
Dataset S12. Untargeted Lipidomics, related to Figure 5
Dataset S13. Differential Gene Expression WT CON v. Med14 S983A CON primary pancreatic beta cells, related to Figure 6
Dataset S14. Differential Gene Expression WT 16h Ex-4 v. WT CON primary pancreatic beta cells, related to Figure 6
Dataset S15. Differential Gene Expression Med14 S983A 16h Ex-4 v. WT CON primary pancreatic beta cells, related to Figure 6
Dataset S16. Differential Gene Expression WT 16h FSK v. WT CON primary pancreatic beta cells, related to Figure 6
Dataset S17. Differential Gene Expression Med14 S983A 16h FSK v. WT CON primary pancreatic beta cells, related to Figure 6

Extended Methods

CRISPR-Cas9 induced targeted mutagenesis and mediator tagging

Guide RNAs were cloned in BsmBI digested LentiCRISPRv2 (Addgene #52961) as described in [5]. For Med14 S983A point mutations, HDR templates with 1kb flanking regions were cloned in pUC19 using the Gibson assembly kit. HDR templates contained Ser983Ala mutations, as well as silent mutations for genotyping (HincII site) and for rendering repaired DNA resistant to Cas9 re-digestion. For mediator tagging, we fused a 3xFLAG/Twin Strep tag to Med19. We selected Med19 based on previous studies in which this subunit was tagged for subsequent purification and cryo-EM structure determination [15]. 1kb Med19-NTD flanking regions were cloned in AAVS1_Puro_PGK1_3xFLAG_Twin_Strep plasmid (Addgene #68375) as described in Dalvai et al. [54]. 2 ug of both LentiCRISPRv2 and repair template plasmid were transfected in INS-1 cells using Maxcyte ExPERT ATx electroporator with Optimization 2 protocol. After transfection, cells were selected in 1 ug/ml puromycin for three days. Single cells were sorted by FACS in 96 well plates. Genomic DNA from resulting clones was extracted with QuickExtract DNA Extraction Solution (Lucigen) for genotyping.

Mediator purification

Mediator was purified as described earlier with some modifications. Briefly, 120 15 cm plates of FLAG-Strep-Med19 knocked-in INS-1 cells were washed with PBS, scraped from the plates and centrifuged at 1,500 rpm. Pellets were resuspended in hypotonic buffer (10 mM HEPES (pH7.9), 1.5mM MgCl2, 10mM KCl, 0.5mM DTT, protease inhibitors, phosphatase inhibitors) and incubated in ice for 15 min. Cell suspension was then transferred to a Dounce homogenizer and homogenized with 15-20 strokes with loose pestle. Next, suspension was centrifuged for 20 min at 20,000 rpm at 4C, followed by a gentle wash of the pellet with another 15 ml of hypotonic buffer and additional centrifugation at 5,000 rpm for 8 min. Pellet was resuspended in twice the pellet volume of extraction buffer (20mM HEPES (pH7.9), 1.5mM MgCl2, 0.6M KCl, 0.2 mM EDTA, 0.5 mM DTT, 25% glycerol, protease inhibitors, phosphatase inhibitors) and rotated at 4C for 1 hour to extract nuclear proteins from chromatin. The nuclear extract was centrifuged for 1 hour at 35,000 rpm, 4C. Cleared supernatant was collected and added to M2 anti-FLAG agarose resin twice pre-washed with equilibration buffer (50mM HEPES (pH7.9), 1.5mM MgCl2, 50mM KCl, 0.3M NaCl) and incubated while rotating at 4C overnight. Beads were then washed with 500 ul elution base buffer (50 mM HEPES (pH7.9), 0.1M NaCl, 1.5mM MgCl2, 0.05% Triton X-100) and eluted with elution base buffer containing FLAG peptide.

Glycerol gradient ultracentrifugation

Nuclear extracts were loaded on top of a 11 ml 10%-30% linear glycerol gradient in gradient buffer (10 mM HEPES pH 7.4, 100 mM KCl, 1.5 mM MgCl2, 100 uM EGTA, 20 mM NaF, 1 mM DTT, protease inhibitors) and centrifuged in a SW41 rotor at 40,000 rpm for 16 hours at 4C. 500ul fractions were collected from the top.

Inducible CREB enhancer identification

For identification of signal responsive CREB bound enhancers, overlapping CREB bound loci from three independent ChIP-seq experiments (1h FSK exposure) were identified using mergePeaks command in Homer. Next, a raw count table of H3AcK27 ChIP-seq tags over these high confidence CREB bound loci (+/- 2kb) from three independent experiments (basal and 1h FSK exposure) was generated using annotatePeaks.pl. Genomic regions with significantly FSK-induced H3AcK27 were identified using DEseq2 (getDiffExpression.pl) (FC FSK/CON >= 1.5, -log adj. p >= 1).

Enhancer activity scoring

Enhancers were scored based on H3K27Ac ChIP-seq data using HOMER (findPeaks <H3K27Ac tag directory> -i <input tag directory> -style super -superSlope -1000 followed by normalization and ranking of enhancer scores.

RNAseq data processing

Raw sequencing reads, including ERCC spike-in controls, were mapped to a combined reference genome comprising the rn6 rat genome and ERCC sequences using STAR (v2.5.3a) with default parameters. Following alignment, quality control and gene quantification were conducted using the 'QoRTs.jar QC' function from the QoRTs (Quality of RNA-Seq ToolSet) software package (v1.3.6) [6]. This process generated raw expression counts for both ERCC spike-ins and rat genes. Gene expression levels were also quantified in transcripts per kilobase million (TPM) across all exons of RefSeq genes using analyzeRepeats.pl in HOMER (v4.11.1) [7], with the top-expressed isoform used as a proxy for gene expression. Differential gene expression analysis was performed using DESeq2 (v1.24.0) [8] based on raw gene counts, incorporating biological replicates to estimate within-group dispersion. ERCC spike-in abundances were used as quality controls to calculate size factors for normalization in DESeq2. Genes were considered differentially expressed with a false discovery rate (FDR) threshold of <0.05 and an absolute fold-change (FC) >1.5 between experimental conditions.

Generation of phospho-Med14S983 antisera

Antisera against phospho-Med14S983 were raised in rabbits against a synthetic phosphopeptide corresponding to Med14(974-991) coupled to maleimide activated keyhole limpet hemocyanin per manufacturer's instructions. The peptide (Cys-DSNQDARRRpSVNEDDNP) was synthesized, HPLC purified and mass spectrometry verified by RS Synthesis (Louisville, KY). The immunogen was prepared by emulsification of Freund's complete adjuvant-modified Mycobacterium butyricum (EMD Millipore) with an equal volume of phosphate buffered saline (PBS) containing 1.0 mg conjugate/ml for the first two injections. For booster injections, incomplete Freund's adjuvant was mixed with an equal volume of PBS containing 0.5 mg conjugate/ml. For each immunization, an animal received a total of 1 ml of emulsion in 20 intradermal sites in the lumbar region, 0.5 mg total protein conjugate for the first two injections and 0.25 mg total protein conjugate for all subsequent booster injections. Three individual rabbits were injected every three weeks and were bled one week following booster injections, <10% total blood volume. Rabbits were administered 1–2 mg/kg Acepromazine IM prior to injections of antigen or blood withdrawal. At the termination of study, rabbits were exsanguinated under anesthesia (ketamine 50 mg/kg and aceprozamine 1 mg/kg, IM) and euthanized with an overdose of pentobarbital sodium and phenytoin sodium (1 ml/4.5 kg of body weight IC to effect). After blood was collected the death of animals was confirmed. All animal procedures were conducted by experienced veterinary technicians, under the supervision of Salk Institute veterinarians.

Affinity purification of phospho-Med14S983 antisera

Antisera were purified using phosphopeptide coupled to SulfoLink coupling resin, 4.6 mg peptide on 2.5 packed bead volume. Coupling was performed by combining peptide and resin in coupling buffer (50 mM Tris, 5 mM EDTA pH 8.5) with 25 mM TCEP and rotating for 1 hour at room temperature followed by alternating washes with 5 column volumes each time of 1 N acetic acid and 50 mM NaHepes, 100 mM NaCl pH 7.5. Next, 20 ml of antiserum was mixed with an equal volume of PBS + 0.02% NaN_3, filtered through 5 um and mixed with drained affinity gel by tumbling overnight at 4C. Gel was washed with 5 column volumes of 10 mM Hepes, pH 7.5 and antibody was eluted with 5 column volumes of 1N Acetic acid.

Oxygen consumption rate (OCR) measurement

INS-1 cells were seeded in XFe 96 microplates at a density of 40,000 cells per well and cultured overnight in RPMI media containing 5 mM glucose. Cells were then washed in serum-free medium without phenol red, sodium bicarbonate, L-glutamine and sodium pyruvate containing 2 mM glucose (Agilent Seahorse XF RPMI Medium, pH 7.4, 103576-100) for 1 hour prior to measurements. Plates were loaded in an XFe 96 Analyzer (Agilent) for OCR measurements. Glucose (20 mM), oligomycin (1 μM) and Antimycin (0.5 μM) + Rotenone (0.5 μM) were injected in the analysis plate at the indicated time points.

Immunofluorescence

Whole mouse pancreas was fixed overnight in Z-fix (Anatech Ltd. 171) and embedded in paraffin. Sections were deparaffinized and rehydrated by incubation in xylenes, 100%, 95%, 70% and 50% ethanol twice for 10 minutes each. For antigen retrieval, sections were then heated at 95°C for 20 minutes and cooled to room temperature in 10 mM sodium citrate (pH 6). Sections were blocked in blocking buffer (PBS/0.3% Triton X-100/5% normal goat serum) for 1 hour at room temperature and exposed to insulin and glucagon antibodies (1/1000 each) overnight in antibody dilution buffer (PBS/1% BSA/0.3% Triton X-100) at 4°C. After three 5 min washes with PBS, sections were incubated with fluorescently labeled anti-guinea pig and anti-rabbit secondary antibodies (1/2000) for one hour at room temperature, washed three times 5 min with PBS and mounted with DAPI Fluoromount-G. Slides were imaged on a Keyence BZ-X710 fluorescence microscope. Images were analyzed for insulin and glucagon-positive area per islet with Fiji [9]. Islets were manually outlined in merged channel images. Insulin and glucagon positive areas in 8-bit images of islets were then tresholded and quantified with the analyze particle tool.

snRNAseq data processing and analysis

FASTQ files were processed using split-pipe (v1.4.0, Parse Biosciences) to generate cell-by-gene count matrices aligned to the mm39 mouse reference genome. Downstream analysis was conducted using Seurat (v5.3.0) [10]. For each sample, nuclei with a number of counts or detected features in the bottom or top 15th percentile were excluded. Additionally, nuclei with >1% mitochondrial gene content were filtered out. Following quality control, individual samples were merged and normalized using SCTransform [11, 12]. Batch effects were corrected using Harmony [13] prior to clustering.

Cluster-specific marker genes were identified using FindAllMarkers, and the top ten markers from each cluster were submitted to CellKb (https://www.cellkb.com) [14] for annotation. To ensure equal representation across samples, the integrated object was downsampled to 2,500 nuclei per sample before downstream analyses. Differential gene expression between treatment groups and across cell types was performed using FindMarkers (min.pct = 0.1, logfc.threshold = 0). Genes were considered significant with an adjusted p value (BH method) less than 0.05. No fold change cutoff

was used. Over-representation analysis was carried out using WebGestaltR (v0.4.6) [15], using the set of all detected genes (prior to filtering) as the reference background.

Insulin secretion assay

Insulin secretion from INS-1 cells was performed as described before [4]. Briefly, A total of 0.5 × 106 INS-1 cells were seeded in a 24-well plate. After 2 days, cells were washed once and incubated in 0.5 ml KRBH buffer (10 mM HEPES [pH 7.4], 5 mM NaHCO3, 129 mM NaCl, 4.8 mM KCl, 1.2 mM KH2PO4, 1.2 mM MgSO4, 1 mM CaCl2) for 2 h to starve cells. Glucose and FSK were added at the indicated concentrations. Buffer was collected and insulin measured with sensitive rat insulin radioimmunoassay (SRI-13K; EMD Millipore) according to the manufacturer's instructions.

Lipid extraction

Lipids were extracted from tissues using the Bligh and Dyer method. Tissues were homogenized in 1:1 phosphate-buffered saline (PBS):methanol, followed by addition of chloroform to achieve a 1:1:2 PBS:methanol:chloroform solvent ratio (v:v:v). Prior to lipid extraction, the following internal standards were added to chloroform unless otherwise stated: SGDG(13C16-16:0/14:0) for quantification of SGDGs and SGAAGs, and ST(d18:1/17:0) for quantification of sulfatides. The mixtures were shaken vigorously for 30 seconds, vortexed for 15 seconds, and centrifuged at 2,200g for 6 minutes at 4°C. The bottom organic layer was collected and dried under a gentle stream of nitrogen.

MS sample preparation and analysis

For proteomics samples were precipitated with trichloroacetic acid (TCA, MP Biomedicals, # 196057) overnight at 4 °C or using Methanol-Chloroform. Dried pellets were dissolved in 8 M urea, reduced with 5 mM tris(2-carboxyethyl) phosphine hydrochloride (TCEP, ThermoFisher, #20491), and alkylated with 10 mM iodoacetamide (Sigma, # I1149). Proteins were then digested overnight at 37 °C with trypsin (Promega, # V5111). The reaction was quenched with formic acid at a final concentration of 5% (v/v). Digested samples were analyzed on a Q Exactive Hybrid Quadrupole-Orbitrap Mass Spectrometer.

Samples were injected directly onto a 25 cm, 100 μm ID column packed with BEH 1.7 μm C18 resin (Waters). Samples were separated at a flow rate of 300 nL/min on an EasynLC 1200 (Thermo). Buffer A and B were 0.1% formic acid in water and 90% acetonitrile, respectively. A gradient of 1–10% B over 30 min, an increase to 35% B over 120 min, an increase to 100% B over 20 min and held at 100% B for a 10 min was used for a 180 min total run time.

Peptides were eluted directly from the tip of the column and nanosprayed directly into the mass spectrometer by application of 2.5 kV voltage at the back of the column. The Eclipse was operated in a data dependent mode. Full MS1 scans were collected in the Orbitrap at 120k resolution. The cycle time was set to 3 s, and within this 3 s the most abundant ions per scan were selected for

CID MS/MS in the ion trap. Monoisotopic precursor selection was enabled and dynamic exclusion was used with exclusion duration of 60 s.

Protein and peptide identification were done with Integrated Proteomics Pipeline – IP2 (Integrated Proteomics Applications). Tandem mass spectra were extracted from raw files using RawConverter [16] and searched with ProLuCID [17] against Uniprot human database. The search space included all fully-tryptic and half-tryptic peptide candidates. Data was searched with 50 ppm precursor ion tolerance and 600 ppm fragment ion tolerance. Identified proteins were filtered to using DTASelect [18] and utilizing a target-decoy database search strategy to control the false discovery rate to 1% at the protein level [19]. Quantitative analysis of TMT was done with Census [20] filtering reporter ions with 10 ppm mass tolerance and 0.6 isobaric purity filter.

Global lipidomics analysis

Lipid extracts, normalized by tissue weight, were injected into a Vanquish UHPLC system coupled to a Q-Exactive Plus mass spectrometer (Thermo Fisher Scientific). Lipids were separated on a Waters XBridge BEH C8 column (5 μm, 50 × 4.6 mm) over a 70-minute gradient: 0% B for 5 minutes, 0-20% B in 0.1 minutes, 20-100% B in 50 minutes, held at 100% B for 8 minutes, 100-0% B in 0.1 minutes, and held at 0% B for 6.9 minutes. The flow rate was initially set to 0.1 mL/min, increased to 0.3 mL/min at 5.1 minutes, and further increased to 0.4 mL/min at 63.1 minutes. Data were acquired in positive and negative ionization modes using Xcalibur.

Solvent A consisted of 95:5 water:methanol, and solvent B consisted of 70:25:5 isopropanol:methanol:water. For positive ionization mode, 0.1% formic acid and 5 mM ammonium formate were added to the mobile phases. For negative ionization mode, 0.1% ammonium hydroxide solution (28% NH3 in water) was added. The HESI source parameters were: spray voltage, 3,000 V (positive mode) and 2,250 V (negative mode); capillary temperature, 325°C; sheath gas, 50; auxiliary gas, 10; spare gas, 1; probe temperature, 200°C; S-Lens RF level, 65.

The Q-Exactive Plus mass spectrometer was operated in data-dependent mode with one full MS scan (resolution 70,000, AGC target 1e6, maximum injection time 100 ms, scan range 150-1,500 m/z), followed by ten higher-energy collisional dissociation (HCD) MS/MS scans (resolution 17,500, AGC target 1 × 105, maximum injection time 200 ms, isolation width 1.0 m/z, stepped normalized collision energy of 20, 30, and 40, scan range 200-2,000 m/z). Dynamic exclusion was set to 30 seconds.

Lipids were identified using LipidSearch (Thermo Fisher Scientific) by comparing MS/MS spectra against a built-in database containing over 1.5 million lipid ions and their predicted fragment ions. LipidSearch parameters were: precursor tolerance, 8 ppm; product tolerance, 10 ppm; positive mode adducts, +H, +NH4, +Na, and +H-H2O; negative mode adducts, −H, −2H, and +Cl; m-Score threshold, 5.0. The ID quality filter was set to A and B, requiring annotation of both head group and fatty acyls for confident identification. Maximum lipid intensity was required to be greater than 1 × 105. Additional manual inspection was performed using Skyline56.

Reagents and Resources

REAGENT OR RESOURCE	SOURCE	IDENTIFIER
Antibodies		

FLAG (mouse)	Sigma	F1804
Phospho-PKA substrate	Cell Signaling	9624
Med12	Cell Signaling	14360
CDK8	Cell Signaling	4106
Med30	Proteintech	67038-1
Phospho-CREB (S133)	Cell Signaling	9198
Fos	Cell Signaling	32154
JunB	Cell Signaling	3753
Phospho-Erk1/2	Cell Signaling	4370
Ras	Cell Signaling	3965
Cpt1a	Abcam	Ab128568
Fasn	Cell Signaling	3180
SREBP-1a	Santa Cruz	Sc-13551
Ldlr	Abcam	Ab30532
tubulin	EMD Millipore	05-829
RNAQ Polymerase II CTD repeat phospho S2	Abcam	Ab5095
Histone H3 (acetyl K27)	Abcam	Ab4729
CRTC2	In house	
phospho-Med14 (S983)	This study	
Insulin	Invitrogen	PA1-26938
Glucagon	Cell Signaling	2760
Alexa Fluor 488 goat anti-guinea pig	Invitrogen	A11073
Alexa Fluor 568 goat anti-rabbit	Invitrogen	A21069
Chemicals, peptides, and recombinant proteins		
Exenatide (Exendin-4)	Bachem	4019602
Semaglutide	Selleck Chemicals	S9697
Tirzepatide	Selleck Chemicals	P1206
FLAG epitope peptide	Bachem	4034775
Anti-FLAG agarose	Sigma	A2220
AMPure XP beads	Beckman Coulter	A63881
Collagenase P	Roche	11213873001
Fetal Bovine lipoprotein deficient serum	Alpha Diagnostic	LDLD47-S
DAPI Fluoromount G	SouthernBiotech	0100-20
Cholesterol	Sigma	C3045
25-dioxycholesterol	Medchemexpress	HY-113134
Recombinant mouse IL-1β	Gibco	PMC0814

Critical commercial assays		
NEBNext Ultra II Directional RNA Library Kit for Illumina	New England Biolabs	E7760
NEBNext Ultra II DNA library Prep Kit for Illumina	New England Biolabs	E7645
Micro BCA protein assay kit	Thermo scientific	23235
Qubit dsDNA HS Assay Kit	Invitrogen	Q32854
Pierce Silver stain kit	Thermo scientific	24612
Transcriptor First Strand cDNA Synthesis Kit	Roche	04897030001
LightCycler480 SYBP Green I Master	Roche	43334520
ERCC RNA Spike-In Mix	Invitrogen	4456740
Phusion Green High-Fidelity DNA Polymerase	Thermo scientific	F-534L
Illumina Tagment DNA Enzyme and Buffer	Illumina	20034210
NEBuilder HiFi DNA Assembly Master Mix	New England Biolabs	E2621
Evercode WT v3	Parse Biosciences	ECWT3300
Evercode Nuclei Fixation v3	Parse Biosciences	ECFN3300
Rat Insulin Radio-immunoassay	EMD Millipore	SRI-13K
Experimental models: Cell lines		
INS-1		
INS-1 Med14 S983A 1	This Study	
INS-1 Med14 S983A 2	This Study	
INS-1 FLAG-MED19	This Study	
INS-1 FLAG-MED19 Med14 S983A	This Study	
HEK 293T		
Experimental models: Organisms/strains		
Med14 S983A mice (C57BL/6J)	This study	
Oligonucleotides		
ATGTCACCTCCTATAGGAGAAGG	Integrated DNA Technologies	Med14G1 (S983A CRISPR guide1)
TGGATAGCAATCAAGATGCTCGG	Integrated DNA Technologies	Med14G2 (S983A CRISPR guide2)
AGATCCCGGGTAACCAAGAGCGG	Integrated DNA Technologies	Med19G1 (CRISPR guide1 for tagging)
GTCAGTTTGAGCTCCGAACAGGG	Integrated DNA Technologies	Med19G2 (CRISPR guide2 for tagging)
GAAAACCAAGCACATGCTGC<br>TTGTTGCACATCAGCAGCAC	Integrated DNA Technologies	Rat *L32* QPCR

CAGCCTTTCCTACTACCATTCC ACAGATCTGCGCAAAAGTCC	Integrated DNA Technologies	Rat *Fos* QPCR
CCTGTTTCCTGTATGGAGGA GGTTCCTCCAAAGGGATATC	Integrated DNA Technologies	Rat *Kdr* QPCR
AGGTGGTGCACCGACTGTTT GGCAAACCTCGTAAAGCCAT	Integrated DNA Technologies	Rat *Osbpl6* QPCR
CCCAGGAGAGCAGTTCTGTC GGATACAGTCTGACGTGAGC	Integrated DNA Technologies	Rat *Plat* QPCR
TTCCAGGTGCACACAGGCTA GCCAGCTCTTTCTGCAGGAT	Integrated DNA Technologies	Rat *iNos* QPCR
GGAGCAAGGTCGCTTACAGA GAAGATAGTAAGCGTGCTCC	Integrated DNA Technologies	Rat *Sod2* QPCR
TGCCCACTCACCCAAGTTCA TGGGTGGTCGGTACAGTGTC	Integrated DNA Technologies	Rat *Ldlr* QPCR
CATCCTCTGTATACGGTTCC CCAGAACCGTAAGAGAACAC	Integrated DNA Technologies	Rat *Hmgcs* QPCR
Recombinant DNA		
pcDNA-Med14	This Study	
pcDNA-Med14 S983A	This Study	
pcDNA-Med14 S992A	This Study	
pcDNA-Med14 S983A/S992A	This Study	
pIRES-PURO3-kRas G12V	This Study	
Software and algorithms		
STAR v2.5.3	Dobin et al. [21]	
MACS2 2.1.2	Zhang et al. [22]	
ATACseqQC 1.20.2	Ou et al. [23]	
TOBIAS 0.14.0	Bentsen et al. [24]	
HOMER 4.11.1	Heinz et al. [7]	
SAMtools 1.9	Li et al. [25]	
QoRTs v1.3.6	Hartley et al. [6]	
DESeq2 1.24.0	Love et al. [8]	

Supplementary Information References

1. Abdella, R., et al., *Structure of the human Mediator-bound transcription preinitiation complex*. Science, 2021. **372**(6537): p. 52-56.
2. Ebmeier, C.C. and D.J. Taatjes, *Activator-Mediator binding regulates Mediator-cofactor interactions*. Proc Natl Acad Sci U S A, 2010. **107**(25): p. 11283-8.
3. Dornbos, P., et al., *Evaluating human genetic support for hypothesized metabolic disease genes*. Cell Metab, 2022. **34**(5): p. 661-666.
4. Van de Velde, S., et al., *CREB Promotes Beta Cell Gene Expression by Targeting Its Coactivators to Tissue-Specific Enhancers*. Mol Cell Biol, 2019. **39**(17).
5. Sanjana, N.E., O. Shalem, and F. Zhang, *Improved vectors and genome-wide libraries for CRISPR screening*. Nat Methods, 2014. **11**(8): p. 783-784.
6. Hartley, S.W. and J.C. Mullikin, *QoRTs: a comprehensive toolset for quality control and data processing of RNA-Seq experiments*. BMC Bioinformatics, 2015. **16**(1): p. 224.
7. Heinz, S., et al., *Simple combinations of lineage-determining transcription factors prime cis-regulatory elements required for macrophage and B cell identities*. Mol Cell, 2010. **38**(4): p. 576-89.
8. Love, M.I., W. Huber, and S. Anders, *Moderated estimation of fold change and dispersion for RNA-seq data with DESeq2*. Genome Biol, 2014. **15**(12): p. 550.
9. Schindelin, J., et al., *Fiji: an open-source platform for biological-image analysis*. Nat Methods, 2012. **9**(7): p. 676-82.
10. Hao, Y., et al., *Dictionary learning for integrative, multimodal and scalable single-cell analysis*. Nat Biotechnol, 2024. **42**(2): p. 293-304.
11. Lause, J., P. Berens, and D. Kobak, *Analytic Pearson residuals for normalization of single-cell RNA-seq UMI data*. Genome Biol, 2021. **22**(1): p. 258.
12. Choudhary, S. and R. Satija, *Comparison and evaluation of statistical error models for scRNA-seq*. Genome Biol, 2022. **23**(1): p. 27.
13. Korsunsky, I., et al., *Fast, sensitive and accurate integration of single-cell data with Harmony*. Nat Methods, 2019. **16**(12): p. 1289-1296.
14. Patil, A.P., A., *CellKb Immune: a manually curated database of mammalian hematopoietic marker gene sets for rapid cell type identification*. bioRxiv, 2020.
15. Elizarraras, J.M., et al., *WebGestalt 2024: faster gene set analysis and new support for metabolomics and multi-omics*. Nucleic Acids Res, 2024. **52**(W1): p. W415-W421.
16. He, L., et al., *Extracting Accurate Precursor Information for Tandem Mass Spectra by RawConverter*. Anal Chem, 2015. **87**(22): p. 11361-7.
17. Xu, T., et al., *ProLuCID: An improved SEQUEST-like algorithm with enhanced sensitivity and specificity*. J Proteomics, 2015. **129**: p. 16-24.

18. Tabb, D.L., W.H. McDonald, and J.R. Yates, 3rd, *DTASelect and Contrast: tools for assembling and comparing protein identifications from shotgun proteomics.* J Proteome Res, 2002. **1**(1): p. 21-6.
19. Peng, J., et al., *Evaluation of multidimensional chromatography coupled with tandem mass spectrometry (LC/LC-MS/MS) for large-scale protein analysis: the yeast proteome.* J Proteome Res, 2003. **2**(1): p. 43-50.
20. Park, S.K., et al., *Census 2: isobaric labeling data analysis.* Bioinformatics, 2014. **30**(15): p. 2208-9.
21. Dobin, A., et al., *STAR: ultrafast universal RNA-seq aligner.* Bioinformatics, 2013. **29**(1): p. 15-21.
22. Zhang, Y., et al., *Model-based analysis of ChIP-Seq (MACS).* Genome Biol, 2008. **9**(9): p. R137.
23. Ou, J., et al., *ATACseqQC: a Bioconductor package for post-alignment quality assessment of ATAC-seq data.* BMC Genomics, 2018. **19**(1): p. 169.
24. Bentsen, M., et al., *ATAC-seq footprinting unravels kinetics of transcription factor binding during zygotic genome activation.* Nat Commun, 2020. **11**(1): p. 4267.
25. Li, H., et al., *The Sequence Alignment/Map format and SAMtools.* Bioinformatics, 2009. **25**(16): p. 2078-9.
